# Global parental acceptance, attitudes, and knowledge regarding human papillomavirus vaccinations for their children: a systematic literature review and meta-analysis

**DOI:** 10.1186/s12905-024-03377-5

**Published:** 2024-09-27

**Authors:** Sophia Heyde, Vanesa Osmani, Gunther Schauberger, Claire Cooney, Stefanie J. Klug

**Affiliations:** TUM School of Medicine and Health, Chair of Epidemiology, Technical University of Munich, Munich, Germany

**Keywords:** Human papillomavirus, Vaccination, Acceptance, Systematic review, Meta-analysis, CRD42019135056

## Abstract

**Background:**

This systematic literature review aims to summarize global research on parental acceptance, attitudes, and knowledge regarding human papillomavirus vaccinations.

**Methods:**

The literature search was conducted in PubMed, Web of Science and Scopus, and included publications from 2006 to 2023. Study quality was assessed using the Newcastle-Ottawa Scale. The Grading of Recommendations Assessment, Development, and Evaluation guidelines were used to assess the strength of evidence for the primary outcome. Meta-analyses were performed using random-effects models to estimate pooled parental acceptance of HPV vaccinations. Studies were stratified by publication years, and a subgroup analysis was conducted to estimate vaccine acceptance rates by world regions. Additionally, sensitivity analyses examined the role of parents in accepting HPV vaccinations for children of different sexes.

**Results:**

Based on 86 studies, we found that parents generally supported HPV vaccinations for their children, yet HPV vaccine acceptance rates showed high variation (12.0 to 97.5%). The subgroup analysis revealed geographical variations in pooled parental HPV vaccine acceptance rates, with the highest rate observed in Africa (79.6%; 95% CI: 73.5–85.2; I² = 98.3%; *p* < 0.01) and the lowest in North America (56.7%; 95% CI: 49.3–64.0; I² = 99.4%; *p* < 0.01). Sensitivity analyses showed that acceptance was higher for daughters than for sons, with mothers more willing to get their daughters vaccinated. The proportion of parents reporting barriers or benefits regarding HPV vaccinations varied widely (0.3 to 95.8%) between study regions. Across all world regions, fear of adverse effects and concerns about vaccine safety were the main barriers, whereas the desire to protect their children from cancer was a significant predictor of vaccine acceptance. Knowledge levels varied widely (6.5 to 100%) between world regions and according to the questions asked. In most studies, knowledge e.g., that HPV is sexually transmitted, and that HPV vaccination provides protection against cervical cancer, ranged from moderate to high.

**Conclusions:**

The results indicated moderate parental acceptance of HPV vaccines. Public knowledge of HPV infection should be promoted, and special efforts should be made to minimize the existing barriers and increase vaccination accessibility and uptake.

**Supplementary Information:**

The online version contains supplementary material available at 10.1186/s12905-024-03377-5.

## Background

### HPV and its impact

Human papillomavirus (HPV) is one of the most common sexually transmitted viruses worldwide [1–4]. In their lifetime, most sexually active individuals will be infected at least once, mostly without developing any pathological changes associated with HPV persistence [5–7]. Over 200 types of HPV are known. Low-risk HPV types 6 and 11 are associated with 90% of genital wart cases, while high-risk HPV types 16 and 18 contribute to 70% of all cervical cancer cases [1, 7]. Worldwide, cervical cancer is the fourth most frequent cancer among women, with an estimated 660,000 new cases and 350,000 deaths in 2022 [7]. Almost all cancer cases are caused by HPV. Additionally, HPV infection is associated with the development of the cancers of the head, neck, anus, and genital tract (i.e., penile, vaginal, and vulvar cancers) [8]. Cervical cancer is considered almost completely preventable due to highly effective primary (HPV vaccine) and secondary (screening) prevention measures [9, 10].

### Global HPV vaccination efforts

The first HPV vaccine was approved in 2006, marking the beginning of HPV vaccination efforts. Currently, there are six licensed HPV vaccines available: three bivalent (Cervarix^®^, Cecolin^®^, Walrinvax^®^, two quadrivalent (Gardasil^®^, Cervavax^®^) and one nonavalent vaccine (Gardasil 9^®^). Since 2009, four of these vaccines have been prequalified by the World Health Organization (Cecolin^®^, Cervarix^®^, Gardasil^®^, Gardasil 9^®^). The bivalent vaccine Walrinvax^®^ is currently under review by the WHO, while the quadrivalent vaccine Cervavac^®^ is licensed for use in specific countries but has not yet received WHO prequalification [11]. The nonavalent HPV vaccine protects against more than 99% of HPV cases related to genotypes 6, 11, 16 and 18 and against up to 96.7% of HPV cases related to genotypes 31, 33, 45, 52, and 58 [12]. As of 2022, 125 countries include HPV vaccine in their routine vaccinations for girls, and 47 countries also for boys [13].

The WHO recommends a one- or two-dose schedule for girls and women between the ages of 9 and 20 years and two doses within a 6-months-interval for women older than 20 years [14]. The strategy of the WHO is to enhance global vaccination programs to increase vaccination rates and incorporate HPV vaccination into national vaccination schemes. As part of this “HPV elimination program”, nationwide vaccination rates of 90% are envisioned by 2030 [15].

### Global parental acceptance, attitudes, and knowledge

Despite the demonstrated high effectiveness against persistent HPV16 and 18 infections, the parental decision to vaccinate their children remains a matter of debate, and HPV vaccination rates in many countries remain low [16–18]. However, global coverage for the first dose of the HPV vaccine in girls grew from 20% in 2022 to 27% in 2023, indicating some progress in vaccination efforts [19]. Research on HPV vaccine acceptance has primarily focused on mothers and daughters, while little is known about the acceptance of HPV vaccines among parents and their sons [20–22]. Parental attitudes and knowledge significantly influence the acceptance of HPV vaccines worldwide. Positive attitudes towards vaccinations in general and specific trust in the efficacy and safety of HPV vaccines are strongly linked to higher acceptance rates [23–25]. Furthermore, increased knowledge about HPV and its link to cervical cancer enhances vaccine acceptance among parents [26–28].

Previous systematic reviews have aimed at addressing the existing research gaps on knowledge, attitudes, and acceptance rates related to the HPV vaccine. Derbie et al. (2023) and Zewdie et al. (2023) examined Ethiopian parents’ attitudes toward vaccinating their children, focusing on local cultural and social factors [29, 30]. Kutz et al. (2023) focused on the awareness and attitudes towards the HPV vaccine in Sub-Saharan Africa, revealing the challenges in promoting vaccination in the region due to limited resources and awareness [31]. López et al. (2020) examined European parental acceptance of HPV vaccines, highlighting the variability in knowledge and acceptance rates across European countries [32]. Suárez et al. (2019) explored the attitudes of Latino fathers in the USA towards the HPV vaccine, pointing out the need for culturally sensitive educational interventions [33]. Perlman et al. (2014) provided a detailed analysis of the knowledge and acceptability of HPV vaccination in Sub-Saharan Africa [34]. Trim et al. (2012) examined parental knowledge and attitudes towards HPV vaccines, emphasizing concerns about safety and information gaps, and how attitudes shifted pre- and post-FDA approval of bivalent and quadrivalent vaccines [35].

Despite these advances in closing knowledge gaps related to HPV vaccines, previous reviews on HPV vaccination had a limited geographic or demographic scope, primarily providing insights into specific regions or populations. This resulted in a lack of a comprehensive global perspective and an incomplete understanding of HPV vaccine acceptance rates, parental attitudes, and knowledge worldwide. For this reason, a comprehensive review and meta-analysis was conducted to qualitatively and quantitatively synthesize existing evidence on parental acceptance, attitudes, and knowledge related to HPV vaccinations for their children. We aimed to (1) quantify the global parental acceptance rates regarding HPV vaccination, (2) identify parental attitudes regarding the perceived benefits and barriers associated with HPV vaccination, and (3) quantify the level of parental knowledge regarding HPV and HPV vaccination.

## Methods

This systematic literature review followed a study protocol registered in the International Prospective Register of Systematic Reviews on July 10, 2019 (PROSPERO, CRD42019135056) and adhered to the Preferred Reporting Items in Systematic Reviews and Meta-Analyses statement [36].

### Literature search

A systematic literature search for quantitative studies was performed in the literature databases PubMed, Web of Science, and Scopus (Additional file 1). Additionally, a hand search of reference lists from relevant studies was conducted to identify any studies that may have been missed in the database search. Covidence was used to remove duplicates and perform title, abstract, and full-text screening. Titles and abstracts of the identified studies were screened for inclusion by the lead author (SH). Studies that met the inclusion criteria were forwarded to full-text screening. Two authors (SH, CC) independently reviewed the full texts of the studies. Any discrepancies or disagreements were discussed until a consensus was reached.

### Inclusion and exclusion criteria

Since HPV vaccines became available in 2006, quantitative studies published online or on paper in English between January 1, 2006 and October 31, 2023 were included. The inclusion criteria were as follows: (1) parental acceptance rates regarding HPV vaccinations (mandatory); (2) parental attitudes regarding HPV vaccinations (optional); (3) parental knowledge of HPV and HPV vaccinations (optional). In the context of this review, “parental” refers to all persons who have legal and/or factual custody of a child. This includes biological parents, adoptive parents, and foster parents. The study participants included parents and their children eligible for HPV vaccinations.

A study was regarded as measuring HPV vaccination acceptance if it evaluated a positive or negative intention or willingness toward vaccinating children in the future (intention to vaccinate) or having consented (already vaccinated) or not to vaccinate their children in the past.

The systematic review excluded studies based on specific criteria, such as studies examining the acceptance of vaccines against sexually transmitted diseases in general. Reviews and meta-analyses were also excluded. Furthermore, studies conducted or published outside the inclusion period, or with populations not matching the demographic criteria, were not considered. This refers specifically to studies asking adults without children to consider hypothetical children, or those exclusively focusing on a pediatric population, or only involving adults. Studies with inappropriate designs, such as qualitative or interventional studies, and those based on convenience or non-probability sampling, were also not included in this review. Additionally, any study that did not report a parental acceptance rate in percent (%) for HPV vaccination for their children was excluded. Non-research materials and studies published in languages other than those specified were also excluded.

### Data extraction

Data were extracted by two independent authors (SH, CC). In case of disagreements, a final decision was reached by consensus. The data extracted included study region, year(s) of study conduct, study setting (refers to the specific location or context in which the study was conducted), parents’ age and sex, children’s age and sex, parents’ ethnicity, the total sample size of parents (n), the survey instrument used, acceptance rate (%), type of acceptance, and sampling method.

### Quality assessment

Study quality was independently assessed by two authors (SH, VO) using the Newcastle-Ottawa Scale (NOS) for cohort studies and a modified version of the NOS for cross-sectional studies (Additional file 2 and 3). In adapting the NOS for cross-sectional studies, we expanded the rating system from 9 to 10 stars to account for the specific methodological differences of this study design.

Disagreements were discussed until a consensus was reached. Each study was rated “very good,” “good,” “satisfactory,” or “unsatisfactory” based on a star system in which a study is judged according to the study group selection, group comparability, and ascertainment of either the exposure or outcome of interest for studies [37, 38].

The quality of the evidence was evaluated using the Grading of Recommendations Assessment, Development, and Evaluation (GRADE) criteria (Additional file 4) [39].

### Data analysis

We descriptively evaluated parental acceptance, the perceived barriers and benefits of HPV vaccinations, and parental knowledge regarding HPV and HPV vaccinations, and presented the findings in tabular form.

Parental acceptance of HPV vaccinations was categorized according to the type of acceptance (intention to vaccinate, already vaccinated, mixed). The proportions of agreement to seven perceived barriers and benefits (desire to protect their children against cancer, recommendation by a pediatrician or family physician, concern regarding vaccine efficacy, concern regarding the adverse effects of HPV vaccines, fear that vaccination will encourage sexual activity, lack of recommendations, and lack of knowledge) were determined. The knowledge level was determined based on the responses to five key HPV-related questions (aware of HPV, aware of HPV vaccines, aware that HPV is sexually transmitted, aware that cervical cancer is related to HPV infection, and aware that HPV vaccines prevent cervical cancer). We summarized the results by world region: (1) Africa; (2) Asia; (3) Australia; (4) Europe; (5) North America; (6) Oceania; (7) South America, as well as by the sex of parents and the sex of children: (1) Parents and daughters; (2) Parents and sons; (3) Parents and children; (4) Mothers and daughters.

### Statistical analysis

A meta-analysis was performed to estimate the overall pooled parental acceptance of HPV vaccination using random-effects models. An additional analysis was conducted to stratify the results by publication year. Only cross-sectional studies that surveyed parents of non-vaccinated children were included. A subgroup analysis was performed based on the world regions. In the sensitivity analyses, we further stratified by the sex of parents and children, as well as by combinations of world region and participant sex.

Statistical heterogeneity among the studies was tested using Cochrane’s Q test (significance level *p* < 0.10). The I^2^ statistic was employed to quantify the heterogeneity of the results using Higgins and Thompson’s guidelines, which indicate that I² values of 25% represent low, 50% medium, and 75% represent high heterogeneity [40, 41]. To ensure robustness, additional analyses for publication bias, small-study effects, and overall effect asymmetry were conducted. Publication bias was assessed using a funnel plot (Additional file 5). Additionally, the trim-and-fill method was used, which adjusts for bias by estimating and imputing the number of missing studies needed to achieve symmetry [42, 43]. Egger’s regression test was used to evaluate the influence of small studies on the overall effect size, with a significant intercept (p-value < 0.05) indicating potential small-study bias [42].

All data analyses were performed using R^®^ (version 4.0.3), using the metafor package (version 3.0.2).

## Results

**Fig. 1:**
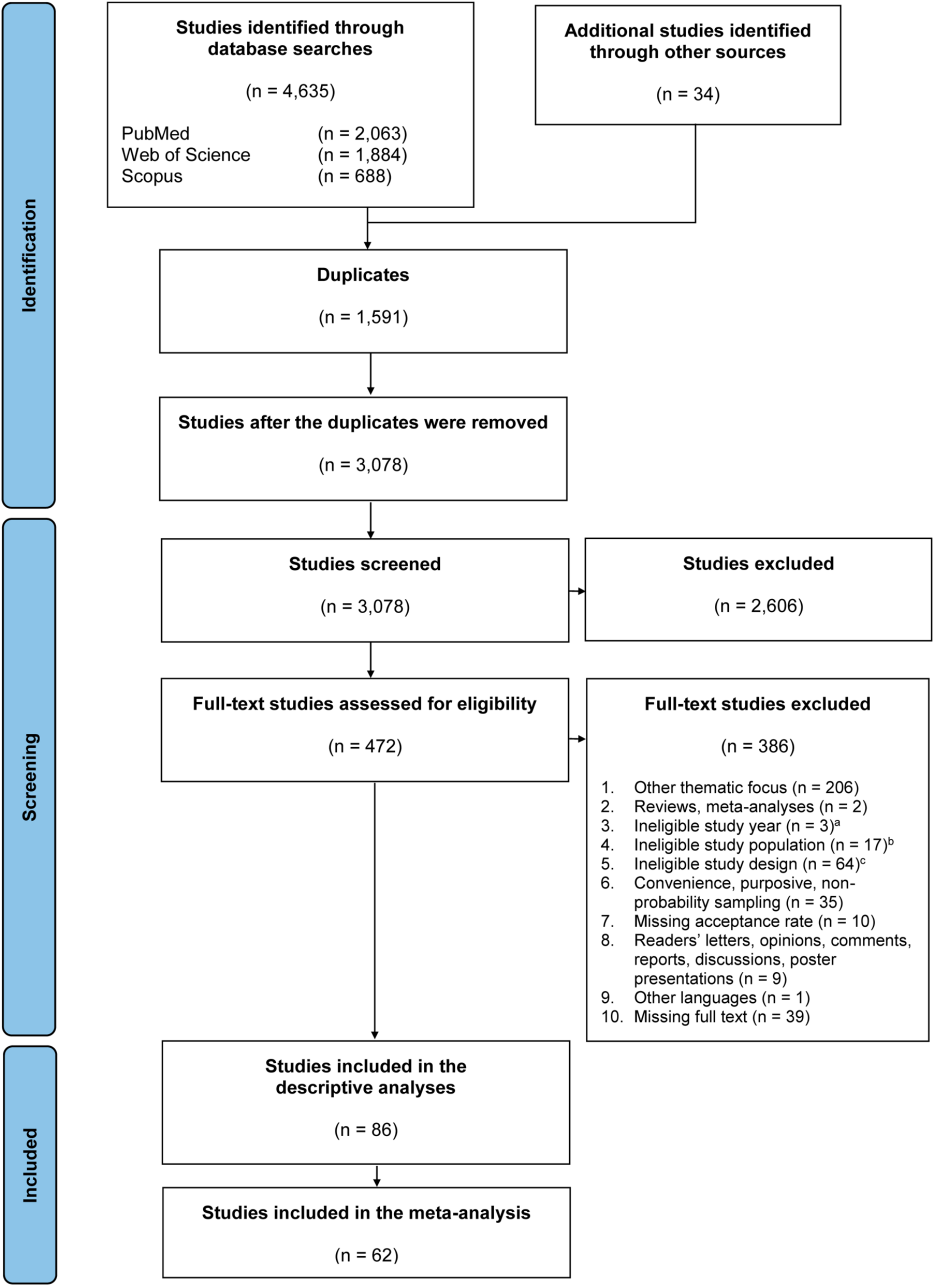
Flow diagram of the study selection process. ^a^ Studies that were conducted or published outside the specified inclusion period from January 1, 2006, to August 31, 2023; ^b^ Studies that did not focus on parents or guardians of children eligible for HPV vaccinations; ^c^ Studies with inappropriate designs (qualitative studies, interventional studies)

We identified 4,635 studies in the literature databases: 2,063 from PubMed, 1,884 from Web of Science, and 688 from Scopus (Fig. 1). Through a hand search, we identified 34 additional studies.

After duplicates were removed, 3,078 studies were reviewed for eligibility, based on the titles and abstracts. According to the exclusion criteria, 2,606 studies were excluded. After reading the full texts of the remaining 472 studies, 386 studies were excluded because they did not meet the inclusion criteria. Thus, 86 studies were included in the systematic literature review, and 62 studies were included in the meta-analysis.

### Study characteristics

We included 83 cross-sectional and three cohort studies representing data from 251,880 parents (Table 1; Additional file 6). The reported study population consisted primarily of parents and their daughters. Simple random sampling was used in more than half of the studies, followed by clustered sampling and stratified random sampling. Most studies reported on the parents’ intention to vaccinate their children. Some studies analyzed the acceptance of HPV vaccinations by parents whose children had been vaccinated at least once. Paper-based questionnaires were used most frequently for data collection, followed by telephone interviews and web-based questionnaires. White parents were the most frequently examined ethnic group, followed by Asians, Blacks, Hispanics and Oceania/Indigenous. The majority of studies included several ethnic groups in their analyses. The studies were mainly conducted in North and South American countries (Canada, USA, Argentina, Brazil). The remaining studies were conducted in European (Austria, Denmark, England, Finland, France, Iceland, Italy, Netherlands, Poland, Romania, Spain, Sweden) and African countries (Ethiopia, Kenya, Morocco, Nigeria, Uganda, Zambia) and the Asia-Pacific Region (China, India, Israel, Korea, Malaysia, Republic of Fiji, Saudi Arabia, Thailand, Vietnam, Australia, New Zealand). Six studies received a study quality rating “very good”, 32 studies “good”, 44 studies “satisfactory”, and four studies “unsatisfactory” (Additional file 7).

**Table 1:** Characteristics of the 86 included studies

	Number of studies (*n*)	Percentage (%)
	86	100
**Study design**		
Cross-sectional	83	96.51
Cohort	3	3.49
**Study participants**		
Parents and children	27	31.40
Parents and daughters	31	36.05
Parents and sons	12	13.95
Mothers and daughters	16	18.60
**Sample size of the parents (n)**		
≤100	2	2.33
101–500	30	34.88
501–999	35	40.70
≥1000	19	22.09
**Sampling method**		
Stratified random sampling	15	17.44
Systematic sampling	4	4.65
Clustered sampling	17	19.77
Simple random sampling	50	58.14
**Year the study was conducted**		
2005–2008	23	26.74
2009–2012	23	26.74
2013–2016	17	19.77
2017–2020	9	10.47
2021–2023	8	9.30
Missing	6	6.98
**Date of study**		
Pre-vaccine licensure^a^	5	5.81
Post-vaccine licensure^a^	81	94.19
**Type of acceptance**		
Intention to vaccinate	63	73.26
Already vaccinated^b^	12	13.95
Mixed^c^	11	12.79
**Survey instrument**		
Computer assisted telephone interview (CATI)	3	3.49
Interviewer-administered questionnaire^d^	11	12.79
Interviewer-administered questionnaire (face-to-face interview)	4	4.65
Interviewer-administered questionnaire (telephone interview)	16	18.60
Paper-based questionnaire	22	25.58
Paper-based questionnaire and face-to-face interview	1	1.16
Paper-based questionnaire and telephone interview	3	3.49
Paper-based questionnaire or web-based questionnaire	1	1.16
Self-administered questionnaire^d^	10	11.63
Web-based questionnaire	15	17.44
**Study region**		
** North America**	**33**	**38.37**
USA	28	32.56
Canada	5	5.81
** Africa**	**19**	**22**,**09**
Ethiopia	7	8.14
Nigeria	4	4.65
Kenya	2	2.33
Uganda	2	2.33
Morocco	1	1.16
Zambia	1	1.16
** Asia**	**15**	**17.44**
China	4	4.65
India	2	2.33
Malaysia	2	2.33
Thailand	2	2.33
Israel	1	1.16
Republic of Fiji	1	1.16
Saudi Arabia	1	1.16
Korea	1	1.16
Vietnam	1	1.16
** Europe**	**15**	**17.44**
Italy	2	2.33
Netherlands	2	2.33
Poland	2	2.33
Austria	1	1.16
Denmark	1	1.16
England	1	1.16
Finland	1	1.16
France	1	1.16
Iceland	1	1.16
Romania	1	1.16
Spain	1	1.16
Sweden	1	1.16
** South America**	**2**	**2.33**
Argentina	1	1.16
Brazil	1	1.16
** Australia**	**1**	**1.16**
** Oceania**	**1**	**1.16**
New Zealand	1	1.16
** Ethnic origin of parents** ^e^		
White	27	31.40
Asian	5	5.81
Black	5	5.81
Hispanic	5	5.81
Oceania/Indigenous	1	1.16
Others	1	1.16
Missing	42	48.84
**Study quality (based on the NOS)**		
Very good	6	6.98
Good	32	37.21
Satisfactory	44	51.16
Unsatisfactory	4	4.65

^a^ Pre-vaccine licensure: 2006 (study period: 2005), post-vaccine licensure: 2007–2023

^b^ Children had already been administered at least one dose of the HPV vaccine

^c^ Intention to vaccinate and already vaccinated

^d^ No information on format

^e^ Studies were classified based on the most frequently studied ethnicity

### Parental acceptance of HPV vaccinations

The HPV vaccine acceptance rates varied widely across the 86 studies (12.0 to 97.5%). Among these, 19 studies reported high acceptance rates (≥ 80%), 62 studies showed moderate acceptance rates (> 30% to < 80%), and five studies reported low acceptance rates (≤ 30%).

Most of the studies involved parents who intended to vaccinate their children (*n* = 62), while in 12 studies, the vaccine had already been administered. Across all 86 studies, sample sizes varied widely, ranging from 39 to 52,855 parents. Studies with high acceptance rates (≥ 80%) and low acceptance rates (≤ 30%) had smaller sample sizes, ranging from 39 to 1,302 and 368 to 1,255 participants, respectively. No obvious trend toward large or small sample sizes was observed. Additionally, studies reporting low acceptance rates exclusively included parents of both sexes. The age of the parents was higher than that in studies with high acceptance rates. In the 86 studies, acceptance rates were higher for daughters than for sons, with mothers more willing to get their daughters vaccinated.

#### Meta-analysis

**Fig. 2:**
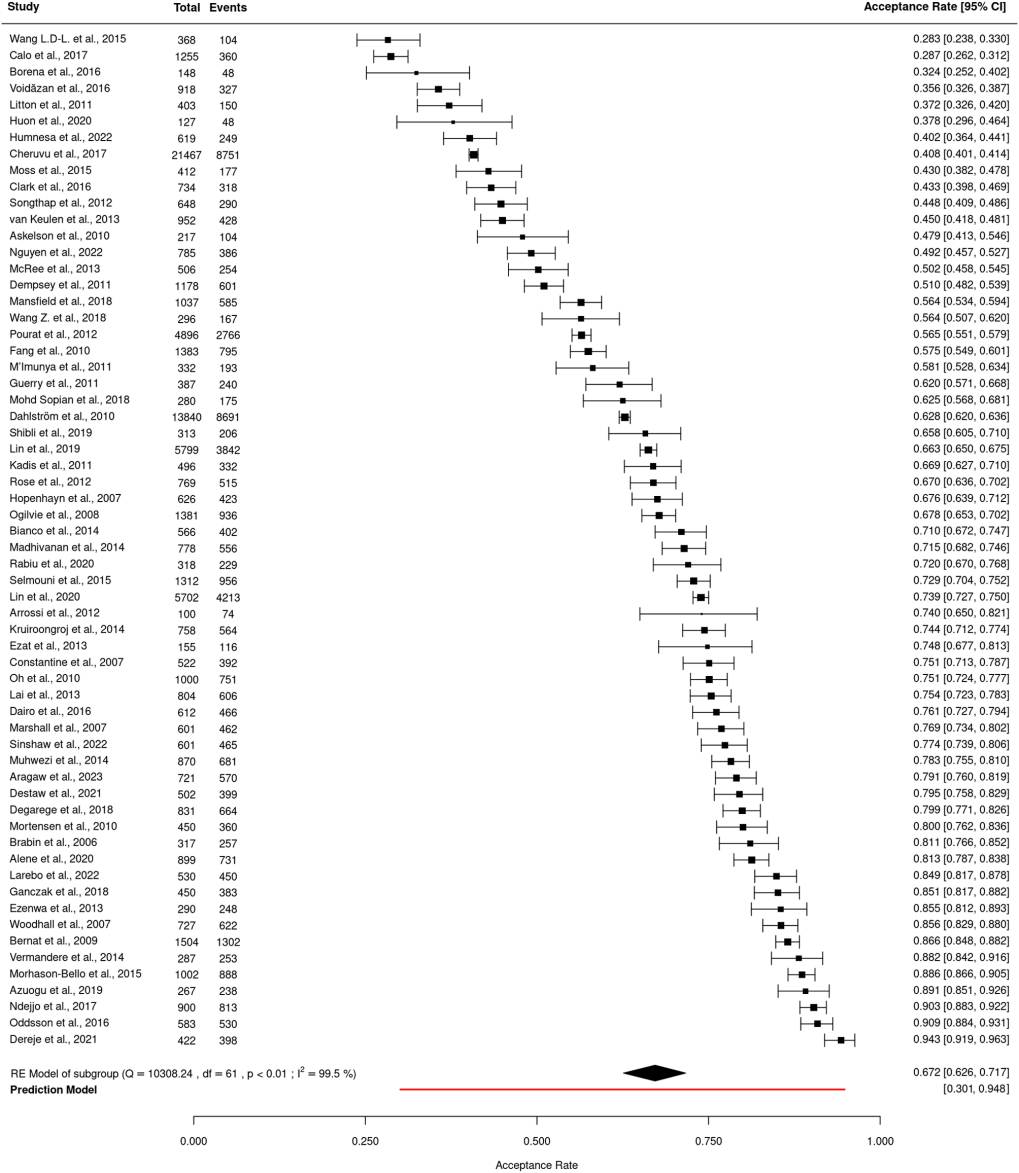
Forest plot for HPV vaccine acceptance rates among parents and their children showing individual study estimates and pooled estimate (random-effects model) (*n* = 62). CI = Confidence interval; df = Degrees of freedom; Events = The number of parents/guardians in each study who reported acceptance of the HPV vaccine for their children; I² = I-squared statistic, indicating the percentage of variation due to heterogeneity; *p* < 0.01 = p-value, indicating statistical significance; Q = Cochran’s Q statistic for heterogeneity; RE Model = Random-effects model; Total = The total number of parents/guardians in each study included in the meta-analysis

For the meta-analysis, 62 cross-sectional studies that surveyed parents of non-vaccinated children were included (Fig. 2). Acceptance rates varied widely across the 62 studies (28.3 to 94.3%) over the years. The pooled acceptance rate for HPV vaccinations across these studies was 67.2% (95% CI: 62.6–71.7; I² = 99.5%; *p* < 0.01; PI: 30.1–94.8), indicating substantial heterogeneity across studies.

Additional analysis stratified by publication year revealed no association in HPV vaccination acceptance rates (Additional file 8). The visual inspection of the funnel plot showed no asymmetry (Additional file 5). The Trim-and-Fill method indicated that no studies were imputed, suggesting no evidence of asymmetry in the data. Egger’s Regression Test for Funnel Plot Asymmetry showed no significant results, confirming the absence of small study bias in the meta-analysis.

Based on the GRADE criteria, the overall vaccine acceptance rate was supported by moderate-quality evidence from 62 studies.

#### Subgroup analysis by world region

**Fig. 3:**
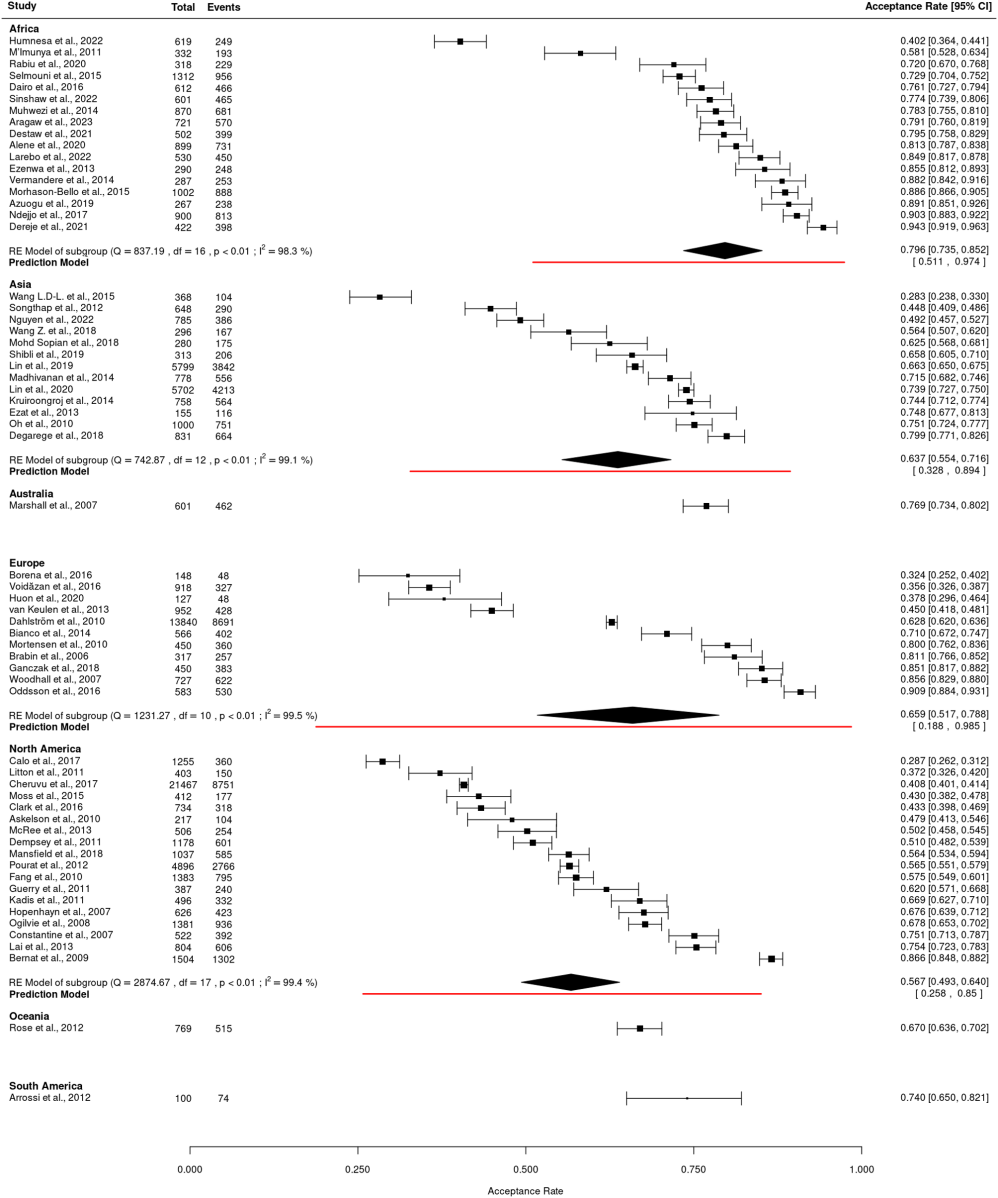
Forest plot for HPV vaccine acceptance rates among parents by world regions showing individual study estimates as well as pooled estimates (random-effects models) (*n* = 62). CI = Confidence interval; df = Degrees of freedom; Events = The number of parents/guardians in each study who reported acceptance of the HPV vaccine for their children; I² = I-squared statistic, indicating the percentage of variation due to heterogeneity; *p* < 0.01 = p-value, indicating statistical significance; Q = Cochran’s Q statistic for heterogeneity; RE Model = Random-effects model; Total = The total number of parents/guardians in each study included in the meta-analysis

Subgroup analysis by world region revealed that studies from Africa had the highest pooled parental acceptance rate for HPV vaccinations (79.6%; 95% CI: 73.5–85.2; I² = 98.3%; *p* < 0.01; PI: 51.1–97.4), followed by studies from Europe (65.9%; 95% CI: 51.7–78.8; I² = 99.5%; *p* < 0.01; PI: 18.8–98.5), Asia (63.7%; 95% CI: 55.4–71.6; I² = 99.1%; *p* < 0.01; PI: 32.8–89.4), and North America (56.7%; 95% CI: 49.3–64.0; I² = 99.4%; *p* < 0.01; PI: 18.8–98.5) (Fig. 3).

The quality of evidence varied across subgroups. For African studies, moderate evidence was observed, while lower quality evidence was found for Asian and North American studies. Studies from Europe presented very low-quality evidence, with a wide PI, highlighting significant uncertainty.

#### Sensitivity analyses by sex of parents and their children

**Fig. 4:**
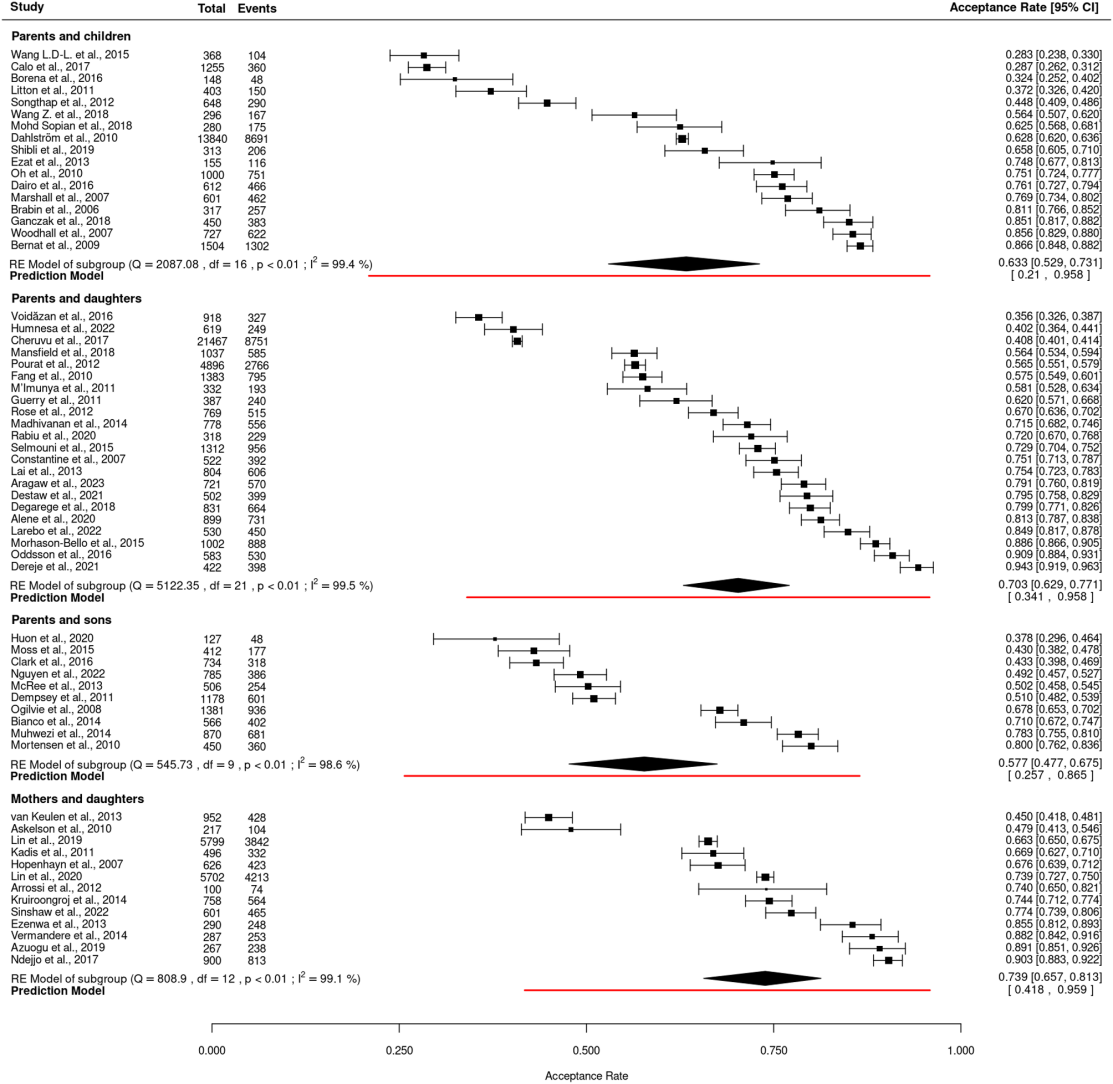
Forest plot for HPV vaccine acceptance rates among parent/children’s subgroups showing individual study estimates as well pooled estimates (random-effects models) (*n* = 62). CI = Confidence interval; df = Degrees of freedom; Events = The number of parents/guardians in each study who reported acceptance of the HPV vaccine for their children; I² = I-squared statistic, indicating the percentage of variation due to heterogeneity; *p* < 0.01 = p-value, indicating statistical significance; Q = Cochran’s Q statistic for heterogeneity; RE Model = Random-effects model; Total = The total number of parents/guardians in each study included in the meta-analysis

The sensitivity analysis by the sex of the parents and their children revealed that the highest pooled acceptance rate was among mothers of daughters (73.9%; 95% CI: 65.7–81.3%; I² = 99.1%; *p* < 0.01; PI: 41.8–95.9), and the lowest was among parents of sons (57.7%; 95% CI: 47.7–67.5%; I² = 98.6%; *p* < 0.01; PI: 25.7–86.5) (Fig. 4). In an analysis stratified by both sex and world regions, the highest rate was among mothers and daughters in studies from Africa (86.3%; 95% CI: 81.6–90.5; I² = 89.4%; *p* < 0.01; PI: 75.5–94.9), and the lowest was among parents and sons in North American studies (51.2%; 95% CI: 42.2–60.2; I² = 97.1%; *p* < 0.01; PI: 29.9–72.3) (Additional file 9).

Sensitivity analyses further underscored challenges, particularly in the “Parents and sons” subgroup, which had very low-quality evidence due to small studies with low study quality. In some cases, publication bias reduced the reliability of findings, notably in the “Parents and daughters, North America”, “Parents and sons, Europe” and “Mothers and daughters, North America” subgroups, which were also impacted by small studies and low study quality.

### Parental attitudes regarding HPV and HPV vaccinations

We extracted data for seven parental barriers and benefits from 43 studies conducted in Africa (*n* = 12), Asia (*n* = 6), Australia (*n* = 1), Europe (*n* = 11), North America (*n* = 10), Oceania (*n* = 1) and South America (*n* = 2) (Table 2).

**Table 2:**
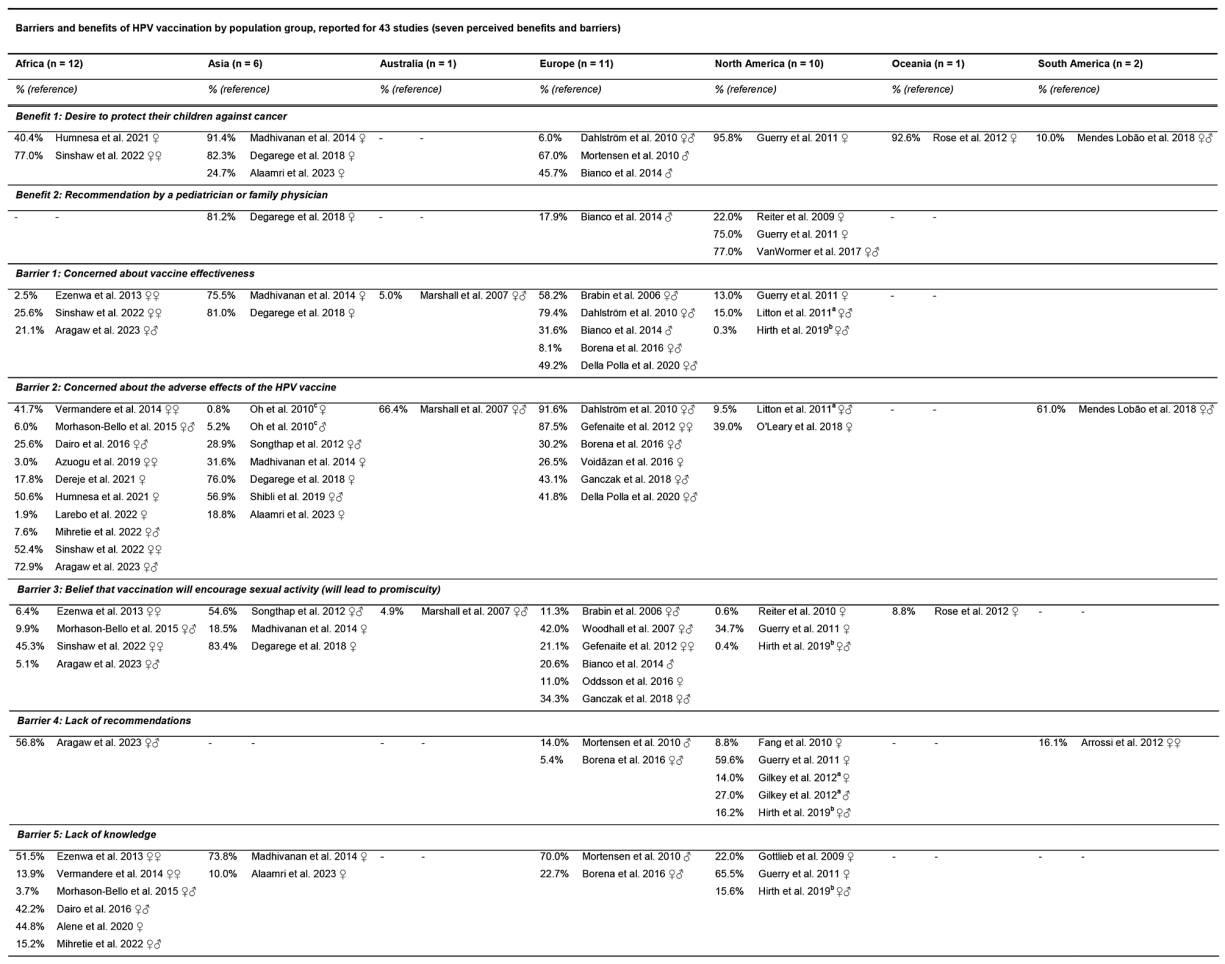
Descriptive analysis of seven parental barriers and benefits to HPV vaccinations reported for 43 studies

♀ = Parents and daughters

♂ = Parents and sons

♀♂ = Parents and children

♀♀ = Mothers and daughters

^a^ Mentioned by intenders

^b^ Mentioned by non-intenders

^c^ Oh et al. 2010 and Gilkey et al. 2012 provided valid percentage values for parents of daughters and parents of sons

In total, 181,736 parents aged 18 to 82 years reported one or several perceived barriers and benefits for parental vaccination intention. Four studies had a study quality rating of “very good”, 14 studies were “good”, 23 studies were “satisfactory”, and two studies were “unsatisfactory”.

The most frequently cited benefit in the studies was the desire to protect children from cancer, while the most frequently cited barrier was concern about the adverse effects of the HPV vaccine.

Regarding benefits, the highest proportion was observed in a study among parents of daughters in North America (USA) and the lowest was noted among European parents of children (Sweden). Regarding barriers, the highest proportion was observed in a study among European parents of children (Sweden) and the lowest was observed among North American parents of children (USA).

#### Benefit 1: Desire to protect their children against cancer

In African studies (Ethiopia; *n* = 2), 40.4 to 77.0% of parents reported cancer protection as a benefit of HPV vaccinations [44, 45], which is higher compared to European studies (Denmark, Italy, Sweden; *n* = 3), where the perception ranged from 6.0 to 67.0% [46–48]. In Asia (India, Saudi Arabia; *n* = 3), the perception of cancer protection as a benefit of HPV vaccination was reported by 24.7 to 91.4% of parents [49–51]. In studies from North America (USA; *n* = 1) and Oceania (New Zealand; *n* = 1) the proportions were higher, with 95.8% and 92.6% of parents, respectively, recognizing this benefit [52, 53]. In contrast, South America (Brazil; *n* = 1) had the lowest reported proportion, with only 10.0% of parents acknowledging cancer protection as a benefit [54].

#### Benefit 2: Recommendation by a pediatrician or family physician

Recommendations by a paediatrician or family physician were most frequently reported as a benefit in studies from North America (USA; *n* = 3), where the proportion ranged from 22.0 to 77.0%, indicating strong influence from healthcare providers [52, 55, 56]. In Asia (India; *n* = 1), a high proportion of 81.2% of parents considered healthcare recommendations a benefit, whereas in Europe (Italy; *n* = 1), only 17.9% of parents shared this view [47, 50].

#### Barrier 1: Concerned about vaccine effectiveness

Parental concern about vaccine effectiveness varied significantly in European studies (England, Italy, Poland, Romania, Sweden; *n* = 5). Most studies (England, Italy, Sweden; *n* = 4) reported moderate rates, ranging from 31.6 to 79.4% [46, 47, 57, 58]. In contrast, studies from North America (USA; *n* = 3) and Africa (Ethiopia, Nigeria; *n* = 3) showed generally low concern, with rates from 0.3 to 15.0% in North America [52, 59, 60] and 2.5 to 25.6% in Africa [45, 61, 62]. In Asian studies (India; *n* = 2), the concern was much higher, ranging from 75.5 to 81.0% [50, 51]. In Australia (*n* = 1), the concern was very low, with only 5.0% of the parents expressing doubts about vaccine efficacy [63].

#### Barrier 2: Concerned about the adverse effects of the HPV vaccine

Concern about the adverse effects of the HPV vaccine was most frequently reported in studies from Africa (Ethiopia, Kenya, Nigeria; *n* = 10), with a wide range of perceptions: six studies indicated lower levels of concern, ranging from 1.9 to 25.6% [28, 64–68], while four studies reported moderate levels, ranging from 41.7 to 72.9% [44, 45, 62, 69]. In Asia (India, Israel, Korea, Saudi Arabia, Thailand; *n* = 6), perceptions also varied, with three studies reporting low concern, ranging from 0.8 to 28.9% [49, 70, 71], and three indicating moderate concern, ranging from 31.6 to 76.0% [50, 51, 72]. In studies from Europe (Austria, Italy, Netherlands, Poland, Romania, Sweden; *n* = 6), parents reported the highest perceived concern on adverse effects, with proportions ranging from 26.5 to 91.6% [25, 46, 57, 73–75]. In North America (USA; *n* = 2), the concern ranged from 9.5 to 39% [60, 76]. In Australia (*n* = 1) and South America (Brazil; *n* = 1), the perceived concern were moderate, at 66.4% and 61.0%, respectively [54, 63].

#### Barrier 3: Belief that vaccination will encourage sexual activity (will lead to promiscuity)

The belief that HPV vaccinations might lead to unprotected sex was most prevalent in European studies (England, Finland, Iceland, Italy, Netherlands, Poland; *n* = 6), with proportions ranging from 11.0 to 42.0% [25, 47, 58, 75, 77, 78]. The concern varied more widely in North American studies (USA; *n* = 3), from 0.4 to 34.7% [52, 59, 79]. This belief was generally less common in studies from Africa (Ethiopia, 371 Nigeria; *n* = 4), with three studies reporting proportions between 6.4 and 9.9% [61, 62, 66]. In Asian studies (India, Thailand; *n* = 3), concern was more pronounced, ranging from 18.5 to 83.4% [50, 51, 71]. In contrast, Australia (*n* = 1) and Oceania (New Zealand; *n* = 1) reported the lowest concern, with 4.9% and 8.8% respectively [53, 63].

#### Barrier 4: Lack of recommendations

Lack of recommendations was most frequently reported as a parental barrier in studies from North America (USA; *n* = 4), where the proportions ranged from 8.8 to 59.6%, indicating significant variation in its influence on vaccination decisions [52, 59, 80, 81]. In European studies (Austria, Denmark; *n* = 2), the range was narrower and lower, from 5.4 to 14.0%, suggesting a more consistent but still notable barrier [48, 74]. In Africa (Ethiopia; *n* = 1), a moderate proportion of 56.8% of parents reported lack of recommendations as a significant barrier, whereas in South America (Argentina; *n* = 1), the proportion was lower at 16.1% [62, 82].

#### Barrier 5: Lack of knowledge

Lack of knowledge was most frequently reported as a parental barrier in studies from Africa (Ethiopia, Kenya, Nigeria; *n* = 6), with proportions ranging from 3.7 to 51.5% [61, 66–69, 83]. In North American studies (USA; *n* = 3), 15.6 to 65.5% of parents reported insufficient knowledge as influencing their decision not to vaccinate their children [52, 59, 84]. In Asian studies (India, Saudi Arabia; *n* = 2), this reason was cited by 10.0 to 73.8% of parents [49, 51]. In studies from Europe (Austria, Denmark; *n* = 2), 22.7 to 70.0% of parents decided against vaccination due to lack of knowledge [48, 74].

### Parental knowledge of HPV and HPV vaccinations

Parental knowledge of HPV and HPV vaccination was assessed as the proportion of the responses to five key HPV-related questions from 52 studies conducted in Africa (*n* = 15), Asia (*n* = 11), Europe (*n* = 10), North America (*n* = 12), Oceania (*n* = 2) and South America (*n* = 2) (Table 3).

**Table 3:**
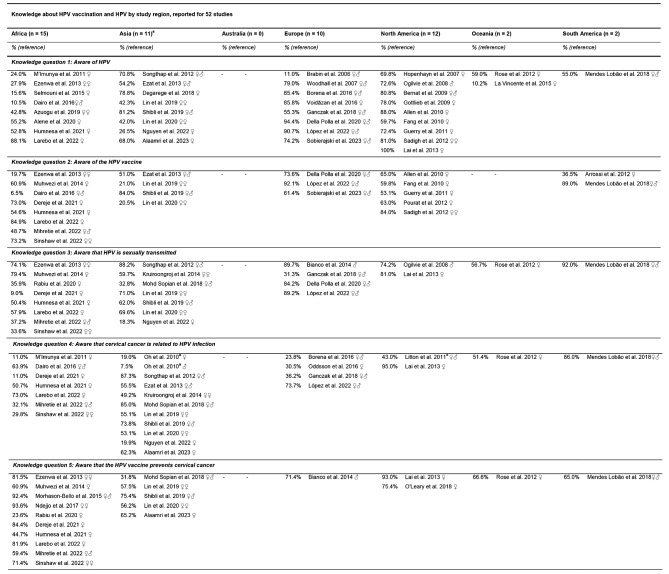
Descriptive analysis of parental knowledge level to HPV vaccinations reported for 52 studies

♀ = Parents and daughters

♂ = Parents and sons

♀♂ = Parents and children

♀♀ = Mothers and daughters

^a^ Oh et al. 2010 provided valid percentage values for parents of daughters and parents of sons

^b^ Mentioned by intenders

In total, 46,905 parents aged 18 to > 70 years answered one or several HPV-related questions. Five studies received a study quality rating of “very good”, 18 studies were “good”, 28 studies were “satisfactory”, and one study was “unsatisfactory”.

The proportion of parental knowledge level across the 52 studies varied widely according to the questions asked (6.5 to 100%), with 19 studies demonstrating high knowledge levels (≥ 80%), 18 studies indicating moderate knowledge levels, and 15 studies showing low knowledge levels (≤ 30%).

Knowledge about HPV was most frequently assessed (*n* = 36), with the highest proportions observed among parents of daughters in a study from North America and the lowest among parents and children in a European study. Following this, awareness that cervical cancer is related to HPV infection was also widely reported (*n* = 25), with the highest proportion found among parents of daughters in a study from North America and the lowest in an Asian study among parents of sons.

#### Knowledge question 1: Aware of HPV

Parental awareness of HPV was highest in North American studies (Canada, USA; *n* = 9), ranging from 59.7 to 100% [52, 80, 84–90], reflecting consistently high awareness across the region. In studies from Africa (Ethiopia, Kenya, Morocco, Nigeria; *n* = 8), awareness varied widely from 10.5 to 88.1%, with higher levels in studies from 2020 onward [44, 61, 64, 65, 68, 83, 91, 92]. Asian studies (China, India, Malaysia, Saudi Arabia, Thailand, Vietnam; *n* = 8) showed similar variation, with rates from 26.5 to 81.2% [49, 50, 71, 72, 93–96]. In European studies (Austria, England, Finland, Italy, Poland, Romania, Spain; *n* = 8), the responses varied from 11.0% to 94.4 [25, 57, 58, 73, 74, 78, 97, 98]. In studies from Oceania (New Zealand, Republic of Fiji; *n* = 2), awareness was 10.2 to 59.0% [53, 99], and in South America (Brazil; *n* = 1), it was 55.0% [54].

#### Knowledge question 2: Aware of the HPV vaccine

Parental awareness of the HPV vaccine was highest in European studies (Italy, Poland, Spain; *n* = 3), ranging from 61.4 to 92.1% [57, 97, 98], showing consistently strong knowledge across the region. North American studies (USA, *n* = 5) also has high awareness, between 53.1 and 84.0% [52, 80, 88, 89, 100]. South American studies (Argentina, Brazil; *n* = 2) showed more variation, with levels from 36.5 to 89.0% [54, 82]. In contrast, studies from Africa (Ethiopia, Nigeria, Uganda; *n* = 8) exhibits significant variation, with awareness ranging from 6.5 to 84.9% [28, 44, 45, 61, 64, 67, 68, 101], indicating lower knowledge in some regions. Asian studies (China, Israel, Malaysia; *n* = 4) also varied widely, with awareness ranging from 20.5 to 84.0% [72, 93–95]. This highlights greater variability in awareness in Africa and Asia compared to the consistently high levels in Europe and North America.

#### Knowledge question 3: Aware that HPV is sexually transmitted

Parental awareness that HPV is sexually transmitted was most consistent in North American studies (Canada, USA; *n* = 2), with levels ranging from 74.2 to 81.0% [86, 90]. European studies (Italy, Poland, Spain; *n* = 4) showed a broader range of awareness, from 31.3 to 89.7%, indicating varying levels of knowledge across the region [25, 47, 57, 97]. In studies from Asia (China, Israel, Malaysia, Thailand, Vietnam; *n* = 7), awareness varies significantly, from low to high, with levels between 18.3 and 88.2%, highlighting considerable differences across countries [71, 72, 94–96, 102, 103]. African studies (Ethiopia, Nigeria, Uganda; *n* = 8) had the widest range, from 9.0 to 79.4%, showing substantial variability in understanding across the region [28, 44, 45, 61, 64, 67, 101, 104]. Oceania (New Zealand; *n* = 1) reports moderate awareness at 56.7% [53], while South America (Brazil; *n* = 1) demonstrates high awareness at 92.0% [54].

#### Knowledge question 4: Aware that cervical cancer is related to HPV infection

Parental awareness of the link between HPV and cervical cancer is highest and most consistent in studies from North America (USA; *n* = 2), ranging from 43.0 to 95.0% [60, 90]. European studies (Austria, Iceland, Poland, Spain; *n* = 4) showed a range of awareness from low to moderate, spanning from 23.8 to 73.7% [25, 74, 77, 97]. Asian studies (China, Israel, Korea, Malaysia, Saudi Arabia, Thailand, Vietnam; *n* = 10) exhibited the widest variability, with awareness ranging from 7.5 to 87.3% [49, 70–72, 93–96, 102, 103]. Similarly, studies from Africa (Ethiopia, Kenya, Nigeria; *n* = 7) showed variability, with ranges from 11.0 to 73.0% [28, 44, 45, 64, 67, 68, 91]. Oceania (New Zealand; *n* = 1) reports moderate awareness at 51.4% [53], and South America (Brazil; *n* = 1) shows high awareness at 86.0% [54].

#### Knowledge question 5: Aware that the HPV vaccine prevents cervical cancer

In African studies (Ethiopia, Nigeria, Uganda; *n* = 10), parental awareness that HPV vaccines prevent cervical cancer ranged from 23.6 to 93.6%, showing significant regional differences [28, 44, 45, 61, 64, 66, 67, 101, 104, 105]. In studies from Asia (China, Israel, Malaysia, Saudi Arabia; *n* = 5), awareness was more consistent, ranging from 31.8 to 75.4% [49, 72, 94, 95, 103]. North American studies (USA; *n* = 2) showed higher awareness levels at 75.4 to 93.0% [76, 90]. Europe (*n* = 1; Italy), Oceania (*n* = 1; New Zealand), and South America (*n* = 1; Brazil) had similar levels of 71.4%, 66.6%, and 65.0%, respectively [47, 53, 54].

## Discussion

This systematic literature review and meta-analysis summarized the global evidence on parental acceptance, attitudes, and knowledge regarding HPV vaccinations.

### Parental acceptance of HPV vaccinations

Parents in the 86 included studies, totaling 251,880, generally supported HPV vaccinations for their children; however, the HPV vaccine acceptance showed a high variation (12 to 97.5%). The acceptance of HPV vaccinations among parents was higher for daughters than for sons, and mothers were more likely to get their daughters vaccinated. This might be explained by insufficient knowledge of the parents on the vaccine’s benefits for male children and adolescents and a perception of the vaccine as a preventative measure against cervical cancer. Sociocultural norms and targeted public health campaigns may also play a role.

The parents who were asked about their intention to vaccinate their children had higher acceptance rates than those whose children had received at least one dose of the HPV vaccine. No study characteristic that could explain the variation in the acceptance rates was identified. Higher acceptance rates in intention-based studies may reflect a general willingness but highlight barriers in translating intent into action, such as logistical challenges and lack of recommendations from healthcare providers.

The acceptance rates for HPV vaccinations in the cohort studies were significantly lower (5.0 to 36.8%) compared to the broader acceptance proportions reported in cross-sectional studies. The lower acceptance rates in these cohort studies, particularly in those that applied the Precaution Adoption Process Model (PAPM), were attributed to their specific focus on parents who had knowledge about the HPV vaccine but still remained hesitant [106, 107].

The meta-analysis showed that acceptance rates varied widely across the 62 studies (28.3 to 94.3%) over the years. The subgroup analysis revealed geographical variations in pooled parental HPV vaccine acceptance rates, with the highest rate observed in Africa and the lowest in North America. This is consistent with the results of other studies, which reported high parental acceptance rates in sub-Saharan Africa because of the available HPV vaccination programs and delivery vaccination strategies [108, 109]. In America, a moderate level of parental intent to have their children vaccinated against HPV was observed. Higher vaccination rates were reported when a healthcare provider recommended HPV vaccination to parents [110–114].

The geographical differences could be explained, potentially due to cultural and health political aspects, on the acceptance rates. Health policies, such as publicly funded national HPV vaccination programs, may enhance HPV acceptance rates. Nonetheless, the high HPV vaccine acceptance rates reported in some studies conducted in Africa or Asia are in contrast to the absence of such policies in these regions [115]. Another geographical factor could be cultural and socioeconomic aspects and attitudes toward vaccination in general. A recent study has demonstrated that low- and middle-income countries show a high acceptance rate of 80.0% for Covid-19 vaccines, whereas, in the USA, it was only 65.0% [116].

It is possible that these differences between low-/middle- and high-income countries are because individuals in high-income countries are more frequently exposed to public information regarding vaccines, safety concerns, side effects, and vaccine-related mortality. Additionally, inhabitants of countries with high availability and easy access to multiple vaccines may become less interested or be more skeptical toward vaccination in general because they may not see the need to receive yet another vaccination and are more exposed to information on vaccine authorization and safety [117].

The acceptance rate could also be correlated with the incidence of HPV in a given region, as the understanding and awareness of HPV could be higher if more individuals are infected. This assumption holds true for the comparison of America and Europe with comparatively lower HPV rates and Africa with the highest worldwide reported HPV rates [118]. Nonetheless, HPV infection rates in Asia are lower than in North America [119, 120], and therefore the high Asian acceptance rates cannot be explained by HPV incidence rates in this region. Moreover, the studies included in the present review do not allow for the conclusion that incidence rates in a given region correlate with acceptance rates, and hence, it remains to be determined to what extent these differences account for the different acceptance rates.

The perceived benefit of HPV vaccinations in preventing cancer and genital warts was the most frequently reported predictor of vaccine acceptance among parents. This is consistent with the findings of other systematic reviews, in which the perceived benefit in preventing cervical cancer not only was rated as the most important attribute of the HPV vaccine but also was positively associated with vaccine acceptance [21, 108]. These reviews had either a narrow population focus or a narrow geographic scope, and evidence on whether and how factors influencing parental HPV vaccine acceptance rates might differ across settings and populations is limited.

While most parents had a positive attitude toward HPV vaccinations, the fear of adverse effects was the most cited reason for vaccine non-acceptance, followed by doubts regarding the effectiveness and safety of the vaccine. This highlights the need for effective communication from health authorities and physicians to address these specific concerns and prevent parental misconceptions. Additionally, studies have confirmed that parents’ belief that HPV vaccinations encourage unprotected sexual intercourse or early sexual activity decreases the likelihood that they will vaccinate their children [121–127]. According to prior research, concerns about the impact of the HPV vaccine on sexual behavior in children are unsubstantiated [128, 129]. Moreover, addressing parents’ concerns on the efficacy and safety of the vaccine could improve vaccination rates. There appears to be a large discrepancy between safety study results and parents’ perception. Providing clear, evidence-based information and engaging healthcare providers in conversations with parents might help bridge this gap [130].

### Parental attitudes regarding HPV and HPV vaccinations

Several studies have identified factors, such as the desire to protect their children against cancer, expectation that HPV vaccination is safe, and the level of knowledge associated with a parent’s acceptance of HPV vaccines and their intention to vaccinate their children [23–25]. Support is generally high (74 to 78%) for a vaccine that protects against genital warts and cervical cancer but varies widely (12 to 100%), depending on the type of vaccine used and parents’ ethnicity [131, 132].

In the US, the highest rates of cervical cancer have been observed among Latin-Americans or African-Americans; however, vaccination rates are suboptimal in these populations. For African-Americans, reasons for not getting their children vaccinated include concerns about the vaccine’s efficacy and safety and their perception of a low infection risk, peer norms, and the perception of discrimination based on their socioeconomic status and their race [132–134]. For Latinos, vaccine acceptance depends primarily on the recommendations of their healthcare provider and the vaccine’s ability to prevent cancer but also on fears of changes in the daughter’s sexual behavior and her fertility as well as on individual and interpersonal reasons [132, 135, 136].

The initiation of HPV vaccinations largely depends on the parental intent to vaccinate their children, which is part of overall vaccine compliance. However, one study on HPV vaccine uptake among adolescents in the USA reported that factors that motivate parents to complete (or not complete) the vaccination series might differ, and the main reason for not intending to complete the HPV vaccine series once initiated was a lack of a recommendation from a healthcare provider for subsequent doses [112]. The parents of previously vaccinated children indicated that having their doctors or healthcare authorities recommend the vaccine increased their likelihood of having their child vaccinated against HPV. These results were consistent with those of previous studies in which practitioner recommendations appeared to be an important factor for vaccination acceptance [137–142]. The absence of a practitioner recommendation is an issue contributing to parental vaccine hesitancy. European studies have reported that nearly a third of hesitant parents never received any HPV vaccine recommendations [143]. It may be concluded that parents seem to trust the recommendations of their healthcare providers, and a lack of such recommendations most likely results in parents hesitating to vaccinate their children and refusing HPV vaccinations.

Theoretical models have identified the intention to vaccinate as an important construct for vaccine-promoting interventions [23, 144]. According to the “Increasing vaccination model”, vaccination rates may be increased by three interventions: modification of thoughts and feelings, motivation through social processes, and leveraging of people’s thoughts and feelings [145]. Good knowledge regarding HPV and cervical cancer and positive attitudes toward vaccines have been associated with higher parental vaccine acceptance and intent to vaccinate [25, 146]. In contrast, barriers to HPV vaccination also affect the vaccine acceptance rates (defined as the willingness to be vaccinated) [135, 136]. Among parents, these barriers include the belief that their child is too young for HPV vaccination, vaccine-related safety concerns, and lack of knowledge [20–22, 110].

### Parental knowledge of HPV and HPV vaccinations

The knowledge level regarding HPV infection and vaccinations varied widely within the population groups. In most studies, the knowledge level regarding HPV, that HPV is sexually transmitted, and HPV vaccinations provide protection against cervical cancer ranged from moderate to high. Several studies have confirmed that parents often express a need for more information regarding HPV vaccinations before deciding whether they should vaccinate their children [140, 147]. A systematic review has identified that insufficient knowledge levels (ranging from 67 to 81%) are a major barrier to HPV vaccinations among parents in Europe [148].

In several studies, parents have reported that they were unaware of the consequences of HPV or had never heard of HPV [60, 132, 149, 150]. However, other studies have indicated that HPV vaccine acceptance increases when parents are well informed about the benefits and risks of HPV vaccinations [24, 25]. A higher maternal level of education has been identified as a significant factor for increasing the acceptance and consequent uptake of HPV vaccinations [146, 151–156].

### Limitations

A strength of this comprehensive review is that 86 studies covering an 18-year period (from 2006 to 2023) with predominantly large sample sizes were included. The main limitation is the considerable heterogeneity between the studies, including differences in the study design and reporting of ethnicity variables. Potential explanations for the residual heterogeneity include variations in the populations from whom the study samples were drawn, different vaccination delivery settings, and selection bias. Because of the differences in the measurements across the included studies and among the population groups, controlling the inconsistencies was difficult. Furthermore, while all eligible studies contained data on parental acceptance of HPV vaccination, not all studies included information on attitudes and knowledge. Although all studies were selected based on defined inclusion and exclusion criteria, the analysis was limited to the parameters that were reported, potentially affecting the comprehensiveness of our findings. We could not derive comprehensive findings on parental acceptance of HPV vaccinations for sons and the acceptance of HPV vaccinations by fathers separately. Overall, the frequent influence of small studies, high heterogeneity, wide PIs, and detected publication bias in certain subgroups underscores the need for cautious interpretation, particularly in cases where the evidence was rated as low or very low quality.

In summary, our findings highlight the importance of addressing the factors promoting HPV vaccine acceptance among parents and their children and the need for evidence-based interventions that address the widespread gaps between HPV vaccine recommendations and actual use [131]. A significant step in advancing acceptance is increasing parental knowledge on HPV, the effects of HPV infection, vaccine safety, and the availability of HPV vaccinations. Specifically targeting those groups of parents who may not be aware of the importance of vaccination in preventing cervical cancer and cancers affecting men, such as parents of sons, could enhance vaccine acceptance and vaccination rates in the future. Future vaccination policies should incorporate the WHO recommendations and consider that a high protection level is already achieved after one injection, which may be more achievable in many countries than a vaccination regimen encompassing two shots.

## Conclusions

Parents’ acceptance of HPV vaccines for their children was moderate in the studies included in this review, with notable variations across world regions. Acceptance rates were highest in Africa, likely due to effective public health campaigns, and lowest in North America, where safety concerns and logistical barriers were more prevalent. Some parents, particularly parents of sons, had limited knowledge on HPV infection and vaccinations, and they were less willing to vaccinate their sons compared to daughters. This suggests a need for targeted education and outreach efforts focused on the benefits of HPV vaccination for boys as well as girls. Given the body of evidence for the safety and effectiveness of HPV vaccinations, public knowledge about HPV infection should be promoted, including efforts to minimize the existing barriers to HPV vaccination and to increase vaccination accessibility and uptake. To counter misinformation and address safety concerns, national campaigns are required. Healthcare providers may support this by informing parents about the safety and benefits of HPV vaccines. Improving parental attitudes toward HPV vaccinations is a key factor for increasing the moderate rates of parental acceptance of such vaccinations. Coordinated efforts among healthcare providers, parents, and government health authorities are urgently required to overcome the barriers to HPV vaccinations and increase parental acceptance.

## Electronic supplementary material

Below is the link to the electronic supplementary material.

Supplementary Material 1

Supplementary Material 2

Supplementary Material 3

Supplementary Material 4

## Data Availability

The datasets used and/or analysed during the current study are available from the corresponding author on reasonable request.

## Abbreviations

CI: Confidence interval
GRADE: Grading of Recommendations Assessment, Development, and Evaluation
HPV: Human papillomavirus
I^2^: Inconsistency index
PI: Prediction interval
PRISMA: Preferred Reporting Items in Systematic Reviews and Meta-Analyses
PROSPERO: Prospective Register of Systematic Reviews
WHO: World Health Organization

## References

[1] Bosch FX, Lorincz A, Muñoz N, Meijer CJLM, Shah KV. The causal relation between human papillomavirus and cervical cancer. J Clin Pathol. 2002;55:244–65. 10.1136/jcp.55.4.244. 11919208 10.1136/jcp.55.4.244 PMC1769629

[2] Herzog TJ. New approaches for the management of cervical cancer. Gynecol Oncol. 2003;90:S22–7. 10.1016/s0090-8258(03)00466-9. 13129492 10.1016/s0090-8258(03)00466-9

[3] Chaturvedi AK. Beyond cervical cancer: burden of other HPV-related cancers among men and women. J Adolesc Health. 2010;46:S20–6. 10.1016/j.jadohealth.2010.01.016. 20307840 10.1016/j.jadohealth.2010.01.016

[4] Saraiya M, Unger ER, Thompson TD, Lynch CF, Hernandez BY, Lyu CW, et al. US assessment of HPV types in cancers: implications for current and 9-valent HPV vaccines. J Natl Cancer Inst. 2015;107:djv086. 10.1093/jnci/djv086. 25925419 10.1093/jnci/djv086 PMC4838063

[5] Chesson HW, Dunne EF, Hariri S, Markowitz LE. The estimated lifetime probability of acquiring human papillomavirus in the United States. Sex Transm Dis. 2014;41:660–4. 10.1097/OLQ.0000000000000193. 25299412 10.1097/OLQ.0000000000000193 PMC6745688

[6] Centers for Disease Control and Prevention (CDC). Human Papillomavirus (HPV): Genital HPV Infection - Fact Sheet. 2017. https://www.cdc.gov/std/hpv/stdfact-hpv.htm. Accessed 3 Mar 2022.

[7] World Health Organization (WHO). Cervical canver: Overview. https://www.who.int/health-topics/cervical-cancer#tab=tab_1. Accessed 20 Aug 2024.

[8] de Martel C, Plummer M, Vignat J, Franceschi S. Worldwide burden of cancer attributable to HPV by site, country and HPV type. Int J Cancer. 2017;141:664–70. 10.1002/ijc.30716. 28369882 10.1002/ijc.30716 PMC5520228

[9] Sung H, Ferlay J, Siegel RL, Laversanne M, Soerjomataram I, Jemal A, Bray F. Global Cancer statistics 2020: GLOBOCAN estimates of incidence and Mortality Worldwide for 36 cancers in 185 countries. CA Cancer J Clin. 2021;71:209–49. 10.3322/caac.21660. 33538338 10.3322/caac.21660

[10] PATH. Projected and current national introductions, demonstration/pilot projects, gender-neutral vaccination programs, and global HPV vaccine introduction maps (2006–2023). 2021. https://www.path.org/resources/global-hpv-vaccine-introduction-overview/. Accessed 3 Mar 2021.

[11] World Health Organization (WHO). Considerations for human papillomavirus (HPV) vaccine product choice: Overview. https://www.who.int/publications/i/item/9789240089167. Accessed 20 Aug 2024.

[12] Petrosky E, Bocchini JA, Hariri S, Chesson H, Curtis CR, Saraiya M, et al. Use of 9-valent human papillomavirus (HPV) vaccine: updated HPV vaccination recommendations of the advisory committee on immunization practices. MMWR Morb Mortal Wkly Rep. 2015;64:300–4. 25811679 PMC4584883

[13] World Health Organization (WHO). Weekly epidemiological record: human papillomavirus vaccines: WHO position paper (2022 update). 2022. https://iris.who.int/bitstream/handle/10665/365350/WER9750-eng-fre.pdf?sequence=1. Accessed 10 Dec 2023.

[14] World Health Organization (WHO). One-dose Human Papillomavirus (HPV) vaccine offers solid protection against cervical cancer. https://www.who.int/news/item/11-04-2022-one-dose-human-papillomavirus-(hpv)-vaccine-offers-solid-protection-against-cervical-cancer. Accessed 31 Oct 2022. PMC9280593 35537734

[15] World Health Organization (WHO). Global strategy to accelerate the elimination of cervical cancer as a public health problem. 2020. https://iris.who.int/bitstream/handle/10665/336583/9789240014107-eng.pdf?sequence=1. Accessed 23 Sep 2024.

[16] Basu P, Malvi SG, Joshi S, Bhatla N, Muwonge R, Lucas E, et al. Vaccine efficacy against persistent human papillomavirus (HPV) 16/18 infection at 10 years after one, two, and three doses of quadrivalent HPV vaccine in girls in India: a multicentre, prospective, cohort study. Lancet Oncol. 2021;22:1518–29. 10.1016/S1470-2045(21)00453-8. 34634254 10.1016/S1470-2045(21)00453-8 PMC8560643

[17] Casey RM, Akaba H, Hyde TB, Bloem P. Covid-19 pandemic and equity of global human papillomavirus vaccination: descriptive study of World Health Organization-Unicef vaccination coverage estimates. BMJ Med. 2024;3:e000726. 10.1136/bmjmed-2023-000726. 38293682 10.1136/bmjmed-2023-000726 PMC10826539

[18] Bergman H, Buckley BS, Villanueva G, Petkovic J, Garritty C, Lutje V, et al. Comparison of different human papillomavirus (HPV) vaccine types and dose schedules for prevention of HPV-related disease in females and males. Cochrane Database Syst Rev. 2019. 10.1002/14651858.CD013479. 31755549 10.1002/14651858.CD013479 PMC6873216

[19] World Health Organization (WHO). Immunization coverage. https://www.who.int/news-room/fact-sheets/detail/immunization-coverage. Accessed 20 Aug 2024.

[20] Zimet GD, Liddon N, Rosenthal SL, Lazcano-Ponce E, Allen B. Chapter 24: Psychosocial aspects of vaccine acceptability. Vaccine. 2006;24 Suppl 3:S3/201-9. 10.1016/j.vaccine.2006.06.017 10.1016/j.vaccine.2006.06.017 16950008

[21] Brewer NT, Fazekas KI. Predictors of HPV vaccine acceptability: a theory-informed, systematic review. Prev Med. 2007;45:107–14. 10.1016/j.ypmed.2007.05.013. 17628649 10.1016/j.ypmed.2007.05.013

[22] Black LL, Zimet GD, Short MB, Sturm L, Rosenthal SL. Literature review of human papillomavirus vaccine acceptability among women over 26 years. Vaccine. 2009;27:1668–73. 10.1016/j.vaccine.2009.01.035. 19195491 10.1016/j.vaccine.2009.01.035

[23] Dempsey AF, Zimet GD, Davis RL, Koutsky L. Factors that are associated with parental acceptance of human papillomavirus vaccines: a randomized intervention study of written information about HPV. Pediatrics. 2006;117:1486–93. 10.1542/peds.2005-1381. 16651301 10.1542/peds.2005-1381

[24] Cheruvu VK, Bhatta MP, Drinkard LN. Factors associated with parental reasons for no-intent to vaccinate female adolescents with human papillomavirus vaccine: National Immunization Survey - Teen 2008–2012. BMC Pediatr. 2017;17:52. 10.1186/s12887-017-0804-1. 28193249 10.1186/s12887-017-0804-1 PMC5307730

[25] Ganczak M, Owsianka B, Korzeń M. Factors that predict parental willingness to have their children vaccinated against HPV in a country with low HPV Vaccination Coverage. Int J Environ Res Public Health. 2018. 10.3390/ijerph15040645. 29614733 10.3390/ijerph15040645 PMC5923687

[26] Paul P, LaMontagne DS, Le NT. Knowledge of cervical cancer and HPV vaccine post- vaccination among mothers and daughters in Vietnam. Asian Pac J Cancer Prev. 2012;13:2587–92. 10.7314/apjcp.2012.13.6.2587. 22938425 10.7314/apjcp.2012.13.6.2587

[27] Grandahl M, Paek SC, Grisurapong S, Sherer P, Tydén T, Lundberg P. Parents’ knowledge, beliefs, and acceptance of the HPV vaccination in relation to their socio-demographics and religious beliefs: a cross-sectional study in Thailand. PLoS ONE. 2018;13:e0193054. 10.1371/journal.pone.0193054. 29447271 10.1371/journal.pone.0193054 PMC5814087

[28] Dereje N, Ashenafi A, Abera A, Melaku E, Yirgashewa K, Yitna M, et al. Knowledge and acceptance of HPV vaccination and its associated factors among parents of daughters in Addis Ababa, Ethiopia: a community-based cross-sectional study. Infect Agent Cancer. 2021;16:58. 10.1186/s13027-021-00399-8. 34479576 10.1186/s13027-021-00399-8 PMC8418033

[29] Derbie A, Mekonnen D, Misgan E, Maier M, Woldeamanuel Y, Abebe T. Acceptance of human papillomavirus vaccination and parents’ willingness to vaccinate their adolescents in Ethiopia: a systematic review and meta-analysis. Infect Agent Cancer. 2023;18:59. 10.1186/s13027-023-00535-6. 37821992 10.1186/s13027-023-00535-6 PMC10566039

[30] Zewdie A, Kasahun AW, Adane HA, Mose A. Willingness to vaccinate their daughters against human papillomavirus among parents of Ethiopian adolescent girls: a systematic review and meta-analysis. J Pharm Policy Pract. 2023;16:126. 10.1186/s40545-023-00639-9. 37875991 10.1186/s40545-023-00639-9 PMC10599018

[31] Kutz J-M, Rausche P, Gheit T, Puradiredja DI, Fusco D. Barriers and facilitators of HPV vaccination in sub-saharan Africa: a systematic review. BMC Public Health. 2023;23:974. 10.1186/s12889-023-15842-1. 37237329 10.1186/s12889-023-15842-1 PMC10214362

[32] López N, Garcés-Sánchez M, Panizo MB, La Cueva IS, Artés MT, Ramos B, Cotarelo M. HPV knowledge and vaccine acceptance among European adolescents and their parents: a systematic literature review. Public Health Rev. 2020;41:10. 10.1186/s40985-020-00126-5. 32435520 10.1186/s40985-020-00126-5 PMC7222509

[33] Suárez P, Wallington SF, Greaney ML, Lindsay AC, Exploring HPV, Knowledge. Awareness, beliefs, attitudes, and vaccine acceptability of latino fathers living in the United States: an integrative review. J Community Health. 2019;44:844–56. 10.1007/s10900-019-00636-7. 30847716 10.1007/s10900-019-00636-7

[34] Perlman S, Wamai RG, Bain PA, Welty T, Welty E, Ogembo JG. Knowledge and awareness of HPV vaccine and acceptability to vaccinate in sub-saharan Africa: a systematic review. PLoS ONE. 2014;9:e90912. 10.1371/journal.pone.0090912. 24618636 10.1371/journal.pone.0090912 PMC3949716

[35] Trim K, Nagji N, Elit L, Roy K. Parental knowledge, attitudes, and Behaviours towards Human Papillomavirus Vaccination for their children: a systematic review from 2001 to 2011. Obstet Gynecol Int. 2012;2012:921236. 10.1155/2012/921236. 21977039 10.1155/2012/921236 PMC3184497

[36] Moher D, Liberati A, Tetzlaff J, Altman DG. Preferred reporting items for systematic reviews and meta-analyses: the PRISMA statement. Ann Intern Med. 2009;151:264–9. 10.7326/0003-4819-151-4-200908180-00135. 19622511 10.7326/0003-4819-151-4-200908180-00135

[37] Wells GA, Shea B, O’Connell D, Peterson J, Welch V, Losos M. Mar, P. Tugwell. The Newcastle-Ottawa Scale (NOS) for assessing the quality of nonrandomised studies in meta-analyses. http://www.ohri.ca/programs/clinical_epidemiology/oxford.asp. Accessed 3 2022.

[38] Herzog R, Álvarez-Pasquin MJ, Díaz C, Del Barrio JL, Estrada JM, Gil Á. Are healthcare workers’ intentions to vaccinate related to their knowledge, beliefs and attitudes? A systematic review. BMC Public Health. 2013;13:154. 10.1186/1471-2458-13-154. 23421987 10.1186/1471-2458-13-154 PMC3602084

[39] Guyatt G, Oxman AD, Akl EA, Kunz R, Vist G, Brozek J, et al. GRADE guidelines: 1. Introduction-GRADE evidence profiles and summary of findings tables. J Clin Epidemiol. 2011;64:383–94. 10.1016/j.jclinepi.2010.04.026. 21195583 10.1016/j.jclinepi.2010.04.026

[40] Higgins JPT, Commentary. Heterogeneity in meta-analysis should be expected and appropriately quantified. Int J Epidemiol. 2008;37:1158–60. 10.1093/ije/dyn204. 18832388 10.1093/ije/dyn204

[41] Higgins JPT, Thompson SG, Deeks JJ, Altman DG. Measuring inconsistency in meta-analyses. BMJ. 2003;327:557–60. 10.1136/bmj.327.7414.557. 12958120 10.1136/bmj.327.7414.557 PMC192859

[42] Quintana DS. From pre-registration to publication: a non-technical primer for conducting a meta-analysis to synthesize correlational data. Front Psychol. 2015;6:1549. 10.3389/fpsyg.2015.01549. 26500598 10.3389/fpsyg.2015.01549 PMC4597034

[43] Lin L, Xu C. Arcsine-based transformations for meta-analysis of proportions: pros, cons, and alternatives. Health Sci Rep. 2020;3:e178. 10.1002/hsr2.178. 32728636 10.1002/hsr2.178 PMC7384291

[44] Humnesa H, Aboma M, Dida N, Abebe M. Knowledge and attitude regarding human papillomavirus vaccine and its associated factors among parents of daughters age between 9–14 years in central Ethiopia, 2021. J Public Health Afr. 2022;13:2129. 10.4081/jphia.2022.2129. 36313923 10.4081/jphia.2022.2129 PMC9614691

[45] Sinshaw MT, Berhe S, Ayele SG. Knowledge and attitude towards Human Papillomavirus Vaccine and Associated factors among mothers who have eligible daughters in Debre Markos Town, Northwest Ethiopia. Infect Drug Resist. 2022;15:781–93. 10.2147/IDR.S352440. 35264861 10.2147/IDR.S352440 PMC8901188

[46] Dahlström LA, Tran TN, Lundholm C, Young C, Sundström K, Sparén P. Attitudes to HPV vaccination among parents of children aged 12–15 years-a population-based survey in Sweden. Int J Cancer. 2010;126:500–7. 10.1002/ijc.24712. 19569173 10.1002/ijc.24712

[47] Bianco A, Pileggi C, Iozzo F, Nobile CGA, Pavia M. Vaccination against human papilloma virus infection in male adolescents: knowledge, attitudes, and acceptability among parents in Italy. Hum Vaccin Immunother. 2014;10:2536–42. 10.4161/21645515.2014.969614. 25483471 10.4161/21645515.2014.969614 PMC4977432

[48] Mortensen GL. Parental attitudes towards vaccinating sons with human papillomavirus vaccine. Dan Med Bull. 2010;57:A4230. 21122463

[49] Alaamri AM, Alghithi AM, Salih S, Omer HM. Acceptance and Associated Risk factors of human papillomavirus vaccine among parents of daughters in Intermediate Schools in Tabuk City, Saudi Arabia. Cureus. 2023;15:e43483. 10.7759/cureus.43483. 37711956 10.7759/cureus.43483 PMC10499461

[50] Degarege A, Krupp K, Fennie K, Srinivas V, Li T, Stephens DP, et al. Human papillomavirus vaccine acceptability among parents of adolescent girls in a rural area, Mysore, India. J Pediatr Adolesc Gynecol. 2018;31:583–91. 10.1016/j.jpag.2018.07.008. 30055285 10.1016/j.jpag.2018.07.008 PMC7679173

[51] Madhivanan P, Li T, Srinivas V, Marlow L, Mukherjee S, Krupp K. Human papillomavirus vaccine acceptability among parents of adolescent girls: obstacles and challenges in Mysore, India. Prev Med. 2014;64:69–74. 10.1016/j.ypmed.2014.04.002. 24732716 10.1016/j.ypmed.2014.04.002

[52] Guerry SL, de Rosa CJ, Markowitz LE, Walker S, Liddon N, Kerndt PR, Gottlieb SL. Human papillomavirus vaccine initiation among adolescent girls in high-risk communities. Vaccine. 2011;29:2235–41. 10.1016/j.vaccine.2011.01.052. 21288799 10.1016/j.vaccine.2011.01.052

[53] Rose SB, Lawton BA, Lanumata TS, Hibma M, Baker MG. Predictors of intent to vaccinate against HPV/cervical cancer: a multi-ethnic survey of 769 parents in New Zealand. N Z Med J. 2012;125:51–62. 22382257

[54] Mendes Lobão W, Duarte FG, Burns JD, de Souza Teles Santos CA, de Almeida C, Reingold MC, Duarte Moreira A. Low coverage of HPV vaccination in the national immunization programme in Brazil: parental vaccine refusal or barriers in health-service based vaccine delivery? PLoS ONE. 2018;13:e0206726. 10.1371/journal.pone.0206726. 30418980 10.1371/journal.pone.0206726 PMC6231618

[55] VanWormer JJ, Bendixsen CG, Vickers ER, Stokley S, McNeil MM, Gee J, et al. Association between parent attitudes and receipt of human papillomavirus vaccine in adolescents. BMC Public Health. 2017;17:766. 10.1186/s12889-017-4787-5. 28969653 10.1186/s12889-017-4787-5 PMC5625818

[56] Reiter PL, Brewer NT, Gottlieb SL, McRee A-L, Smith JS. Parents’ health beliefs and HPV vaccination of their adolescent daughters. Soc Sci Med. 2009;69:475–80. 10.1016/j.socscimed.2009.05.024. 19540642 10.1016/j.socscimed.2009.05.024

[57] Della Polla G, Pelullo CP, Napolitano F, Angelillo IF. HPV vaccine hesitancy among parents in Italy: a cross-sectional study. Hum Vaccin Immunother. 2020;16:2744–51. 10.1080/21645515.2020.1744367. 32298210 10.1080/21645515.2020.1744367 PMC7734096

[58] Brabin L, Roberts SA, Farzaneh F, Kitchener HC. Future acceptance of adolescent human papillomavirus vaccination: a survey of parental attitudes. Vaccine. 2006;24:3087–94. 10.1016/j.vaccine.2006.01.048. 16500736 10.1016/j.vaccine.2006.01.048

[59] Hirth JM, Fuchs EL, Chang M, Fernandez ME, Berenson AB. Variations in reason for intention not to vaccinate across time, region, and by race/ethnicity, NIS-Teen (2008–2016). Vaccine. 2019;37:595–601. 10.1016/j.vaccine.2018.12.017. 30580838 10.1016/j.vaccine.2018.12.017 PMC6559359

[60] Litton AG, Desmond RA, Gilliland J, Huh WK, Franklin FA. Factors associated with intention to vaccinate a daughter against HPV: a statewide survey in Alabama. J Pediatr Adolesc Gynecol. 2011;24:166–71. 10.1016/j.jpag.2011.01.004. 21397534 10.1016/j.jpag.2011.01.004 PMC3100399

[61] Ezenwa BN, Balogun MR, Okafor IP. Mothers’ human papilloma virus knowledge and willingness to vaccinate their adolescent daughters in Lagos, Nigeria. Int J Womens Health. 2013;5:371–7. 10.2147/IJWH.S44483. 23874123 10.2147/IJWH.S44483 PMC3711756

[62] Aragaw GM, Anteneh TA, Abiy SA, Bewota MA, Aynalem GL. Parents’ willingness to vaccinate their daughters with human papillomavirus vaccine and associated factors in Debretabor town, Northwest Ethiopia: a community-based cross-sectional study. Hum Vaccin Immunother. 2023;19:2176082. 10.1080/21645515.2023.2176082. 36794293 10.1080/21645515.2023.2176082 PMC10026865

[63] Marshall H, Ryan P, Roberton D, Baghurst P. A cross-sectional survey to assess community attitudes to introduction of human papillomavirus vaccine. Aust N Z J Public Health. 2007;31:235–42. 17679241 10.1111/j.1467-842x.2007.00054.x

[64] Larebo YM, Elilo LT, Abame DE, Akiso DE, Bawore SG, Anshebo AA, Gopalan N. Awareness, Acceptance, and Associated factors of human papillomavirus vaccine among parents of daughters in Hadiya Zone, Southern Ethiopia: a cross-sectional study. Vaccines (Basel). 2022;10:1988. 10.3390/vaccines10121988. 36560398 10.3390/vaccines10121988 PMC9785952

[65] Azuogu BN, Umeokonkwo CD, Azuogu VC, Onwe OE, Okedo-Alex IN, Egbuji CC. Appraisal of willingness to vaccinate daughters with human papilloma virus vaccine and cervical cancer screening uptake among mothers of adolescent students in Abakaliki, Nigeria. Niger J Clin Pract. 2019;22:1286–91. 10.4103/njcp.njcp_452_18. 31489868 10.4103/njcp.njcp_452_18

[66] Morhason-Bello IO, Wallis S, Adedokun BO, Adewole IF. Willingness of reproductive-aged women in a Nigerian community to accept human papillomavirus vaccination for their children. J Obstet Gynaecol Res. 2015;41:1621–9. 10.1111/jog.12775. 26310912 10.1111/jog.12775

[67] Mihretie GN, Liyeh TM, Ayele AD, Belay HG, Yimer TS, Miskr AD. Knowledge and willingness of parents towards child girl HPV vaccination in Debre Tabor Town, Ethiopia: a community-based cross-sectional study. Reprod Health. 2022;19:136. 10.1186/s12978-022-01444-4. 35689288 10.1186/s12978-022-01444-4 PMC9188100

[68] Dairo MD, Adeleke MO, Salawu AT, Adewole AD. Parental support for human papilloma virus vaccination by adolescents in Ibadan North Local Government Area, Ibadan, Nigeria. Int J Adolesc Med Health. 2016. 10.1515/ijamh-2016-0034. 27914213 10.1515/ijamh-2016-0034

[69] Vermandere H, Naanyu V, Mabeya H, Vanden Broeck D, Michielsen K, Degomme O. Determinants of acceptance and subsequent uptake of the HPV vaccine in a cohort in Eldoret, Kenya. PLoS ONE. 2014;9:e109353. 10.1371/journal.pone.0109353. 25299646 10.1371/journal.pone.0109353 PMC4192319

[70] Oh J-K, Lim MK, Yun EH, Lee E-H, Shin H-R. Awareness of and attitude towards human papillomavirus infection and vaccination for cervical cancer prevention among adult males and females in Korea: a nationwide interview survey. Vaccine. 2010;28:1854–60. 10.1016/j.vaccine.2009.11.079. 20005860 10.1016/j.vaccine.2009.11.079

[71] Songthap A, Pitisuttithum P, Kaewkungwal J, Fungladda W, Bussaratid V. Knowledge, attitudes, and acceptability of a human papilloma virus vaccine among students, parents and teachers in Thailand. Southeast Asian J Trop Med Public Health. 2012;43:340–53. 23082586

[72] Shibli R, Rishpon S. The factors associated with maternal consent to human papillomavirus vaccination among adolescents in Israel. Hum Vaccin Immunother. 2019;15:3009–15. 10.1080/21645515.2019.1631139. 31339452 10.1080/21645515.2019.1631139 PMC6930114

[73] Voidăzan S, Tarcea M, Morariu S-H, Grigore A, Dobreanu M. Human papillomavirus vaccine - knowledge and attitudes among parents of children aged 10–14 years: a cross-sectional study, Tîrgu Mureş, Romania. Cent Eur J Public Health. 2016;24:29–38. 10.21101/cejph.a4287. 27070967 10.21101/cejph.a4287

[74] Borena W, Luckner-Hornischer A, Katzgraber F, Holm-von Laer D. Factors affecting HPV vaccine acceptance in West Austria: do we need to revise the current immunization scheme? Papillomavirus Res. 2016;2:173–7. 10.1016/j.pvr.2016.10.001. 29074178 10.1016/j.pvr.2016.10.001 PMC5886907

[75] Gefenaite G, Smit M, Nijman HW, Tami A, Drijfhout IH, Pascal A, et al. Comparatively low attendance during human papillomavirus catch-up vaccination among teenage girls in the Netherlands: insights from a behavioral survey among parents. BMC Public Health. 2012;12:498. 10.1186/1471-2458-12-498. 22748022 10.1186/1471-2458-12-498 PMC3461412

[76] O’Leary ST, Lockhart S, Barnard J, Furniss A, Dickinson M, Dempsey AF, et al. Exploring facilitators and barriers to initiation and completion of the human papillomavirus (HPV) Vaccine Series among parents of girls in a Safety Net System. Int J Environ Res Public Health. 2018. 10.3390/ijerph15020185. 29360785 10.3390/ijerph15020185 PMC5858260

[77] Oddsson K, Gudmundsdottir T, Briem H. Attitudes and knowledge among parents or guardians of 12-year-old girls about HPV vaccination - A population-based survey in Iceland. Eur J Gynaecol Oncol. 2016;37:837–41. 29943932

[78] Woodhall SC, Lehtinen M, Verho T, Huhtala H, Hokkanen M, Kosunen E. Anticipated acceptance of HPV vaccination at the baseline of implementation: a survey of parental and adolescent knowledge and attitudes in Finland. J Adolesc Health. 2007;40:466–9. 10.1016/j.jadohealth.2007.01.005. 17448408 10.1016/j.jadohealth.2007.01.005

[79] Reiter PL, Cates JR, McRee A-L, Gottlieb SL, Shafer A, Smith JS, Brewer NT. Statewide HPV vaccine initiation among adolescent females in North Carolina. Sex Transm Dis. 2010;37:549–56. 10.1097/OLQ.0b013e3181d73bf8. 20414146 10.1097/OLQ.0b013e3181d73bf8 PMC4018582

[80] Fang CY, Coups EJ, Heckman CJ. Behavioral correlates of HPV vaccine acceptability in the 2007 Health Information National trends Survey (HINTS). Cancer Epidemiol Biomarkers Prev. 2010;19:319–26. 10.1158/1055-9965.EPI-09-0918. 20142234 10.1158/1055-9965.EPI-09-0918 PMC2820128

[81] Gilkey MB, Moss JL, McRee A-L, Brewer NT. Do correlates of HPV vaccine initiation differ between adolescent boys and girls? Vaccine. 2012;30:5928–34. 10.1016/j.vaccine.2012.07.045. 22841973 10.1016/j.vaccine.2012.07.045 PMC3438656

[82] Arrossi S, Maceira V, Paolino M, Sankaranarayanan R. Acceptability and uptake of HPV vaccine in Argentina before its inclusion in the immunization program: a population-based survey. Vaccine. 2012;30:2467–74. 10.1016/j.vaccine.2012.01.032. 22266289 10.1016/j.vaccine.2012.01.032

[83] Alene T, Atnafu A, Mekonnen ZA, Minyihun A. Acceptance of human papillomavirus vaccination and associated factors among parents of daughters in Gondar Town, Northwest Ethiopia. Cancer Manage Res. 2020;12:8519–26. 10.2147/CMAR.S275038. 10.2147/CMAR.S275038 PMC7502398 32982444

[84] Gottlieb SL, Brewer NT, Sternberg MR, Smith JS, Ziarnowski K, Liddon N, Markowitz LE. Human papillomavirus vaccine initiation in an area with elevated rates of cervical cancer. J Adolesc Health. 2009;45:430–7. 10.1016/j.jadohealth.2009.03.029. 19837348 10.1016/j.jadohealth.2009.03.029

[85] Hopenhayn C, Christian A, Christian WJ, Schoenberg NE. Human papillomavirus vaccine: knowledge and attitudes in two Appalachian Kentucky counties. Cancer Causes Control. 2007;18:627–34. 10.1007/s10552-007-9007-7. 17497223 10.1007/s10552-007-9007-7

[86] Ogilvie GS, Remple VP, Marra F, McNeil SA, Naus M, Pielak K, et al. Intention of parents to have male children vaccinated with the human papillomavirus vaccine. Sex Transm Infect. 2008;84:318–23. 10.1136/sti.2007.029389. 18445636 10.1136/sti.2007.029389

[87] Bernat DH, Harpin SB, Eisenberg ME, Bearinger LH, Resnick MD. Parental support for the human papillomavirus vaccine. J Adolesc Health. 2009;45:525–7. 10.1016/j.jadohealth.2009.04.014. 19837360 10.1016/j.jadohealth.2009.04.014

[88] Allen JD, Othus MKD, Shelton RC, Li Y, Norman N, Tom L, del Carmen MG. Parental decision making about the HPV vaccine. Cancer Epidemiol Biomarkers Prev. 2010;19:2187–98. 10.1158/1055-9965.EPI-10-0217. 20826829 10.1158/1055-9965.EPI-10-0217

[89] Sadigh G, Dempsey AF, Ruffin M, Resnicow K, Carlos RC. National patterns in human papillomavirus vaccination: an analysis of the National Survey of Family Growth. Hum Vaccin Immunother. 2012;8:234–42. 10.4161/hv.18456. 22414967 10.4161/hv.18456

[90] Lai JY, Tinker AV, Cheung WY. Factors influencing the willingness of US women to vaccinate their daughters against the human papillomavirus to prevent cervical cancer. Med Oncol. 2013;30:582. 10.1007/s12032-013-0582-z. 23609191 10.1007/s12032-013-0582-z

[91] M’Imunya JM, Ogutu O. Parental Acceptance of Human Papilloma Virus Vaccine for their pre-pubertal and teenage daughters. E Af Med Jrnl. 2011;88:163–70.

[92] Selmouni F, Zidouh A, Nejjari C, Bekkali R. Acceptability of the human papilloma virus vaccine among Moroccan parents: a population-based crosssectional study. East Mediterr Health J. 2015;21:555–63. 26446526 10.26719/2015.21.8.555

[93] Ezat SWP, Hod R, Mustafa J, Mohd Dali AZH, Sulaiman AS, Azman A. National HPV immunisation programme: knowledge and acceptance of mothers attending an obstetrics clinic at a teaching hospital, Kuala Lumpur. Asian Pac J Cancer Prev. 2013;14:2991–9. 10.7314/APJCP.2013.14.5.2991. 23803068 10.7314/apjcp.2013.14.5.2991

[94] Lin W, Wang Y, Liu Z, Chen B, Yuan S, Wu B, Gong L. Awareness and attitude towards human papillomavirus and its vaccine among females with and without daughter(s) who participated in cervical cancer screening in Shenzhen, China. Trop Med Int Health. 2019;24:1054–63. 10.1111/tmi.13283. 31264319 10.1111/tmi.13283

[95] Lin W, Zhou L, Hu H, Chen B, Yuan S, Wu B, et al. The number and gender of children synergistically impact on a mother’s practice of human papillomavirus testing and attitudes towards vaccination in Shenzhen, China. Cancer Epidemiol. 2020;65:101682. 10.1016/j.canep.2020.101682. 32036245 10.1016/j.canep.2020.101682

[96] Nguyen LH, Le TBT, Le NQN, Tran NTT. Acceptance and willingness to pay for Vaccine Against Human Papilloma Virus (HPV) among parents of boys in Central Vietnam. Front Public Health. 2022;10:801984. 10.3389/fpubh.2022.801984. 35356024 10.3389/fpubh.2022.801984 PMC8960026

[97] López N, La Cueva IS, Taborga E, de Alba AF, Cabeza I, Raba RM, et al. HPV knowledge and vaccine acceptability: a survey-based study among parents of adolescents (KAPPAS study). Infect Agent Cancer. 2022;17:55. 10.1186/s13027-022-00467-7. 36397080 10.1186/s13027-022-00467-7 PMC9670070

[98] Sobierajski T, Małecka I, Augustynowicz E. Feminized vaccine? Parents’ attitudes toward HPV vaccination of adolescents in Poland: a representative study. Hum Vaccin Immunother. 2023;19:2186105. 10.1080/21645515.2023.2186105. 36949646 10.1080/21645515.2023.2186105 PMC10064925

[99] La Vincente SF, Mielnik D, Jenkins K, Bingwor F, Volavola L, Marshall H, et al. Implementation of a national school-based human papillomavirus (HPV) vaccine campaign in Fiji: knowledge, vaccine acceptability and information needs of parents. BMC Public Health. 2015;15:1257. 10.1186/s12889-015-2579-3. 26684658 10.1186/s12889-015-2579-3 PMC4684606

[100] Pourat N, Jones JM. Role of insurance, income, and affordability in human papillomavirus vaccination. Am J Manag Care. 2012;18:320–30. 22775000

[101] Muhwezi WW, Banura C, Turiho AK, Mirembe F. Parents’ knowledge, risk perception and willingness to allow young males to receive human papillomavirus (HPV) vaccines in Uganda. PLoS ONE. 2014;9:e106686. 10.1371/journal.pone.0106686. 25203053 10.1371/journal.pone.0106686 PMC4159277

[102] Kruiroongroj S, Chaikledkaew U, Thavorncharoensap M. Knowledge, acceptance, and willingness to pay for human papilloma virus (HPV) vaccination among female parents in Thailand. Asian Pac J Cancer Prev. 2014;15:5469–74. 10.7314/apjcp.2014.15.13.5469. 25041020 10.7314/apjcp.2014.15.13.5469

[103] Mohd Sopian M, Shaaban J, Mohd Yusoff SS, Wan Mohamad WMZ, Knowledge. Decision-making and Acceptance of Human Papilloma Virus Vaccination among Parents of Primary School Students in Kota Bharu, Kelantan, Malaysia. Asian Pac J Cancer Prev. 2018;19:1509–14. 10.22034/APJCP.2018.19.6.1509. 29936724 10.22034/APJCP.2018.19.6.1509 PMC6103591

[104] Rabiu KA, Alausa TG, Akinlusi FM, Davies NO, Shittu KA, Akinola OI. Parental acceptance of human papillomavirus vaccination for adolescent girls in Lagos, Nigeria. J Family Med Prim Care. 2020;9:2950–7. 10.4103/jfmpc.jfmpc_102_20. 32984154 10.4103/jfmpc.jfmpc_102_20 PMC7491808

[105] Ndejjo R, Mukama T, Musinguzi G, Halage AA, Ssempebwa JC, Musoke D. Women’s intention to screen and willingness to vaccinate their daughters against cervical cancer - a cross sectional study in eastern Uganda. BMC Public Health. 2017;17:255. 10.1186/s12889-017-4180-4. 28288614 10.1186/s12889-017-4180-4 PMC5348782

[106] Tatar O, Shapiro GK, Perez S, Wade K, Rosberger Z. Using the precaution adoption process model to clarify human papillomavirus vaccine hesitancy in Canadian parents of girls and parents of boys. Hum Vaccin Immunother. 2019;15:1803–14. 10.1080/21645515.2019.1575711. 30735442 10.1080/21645515.2019.1575711 PMC6746468

[107] Perez S, Tatar O, Gilca V, Shapiro GK, Ogilvie G, Guichon J, et al. Untangling the psychosocial predictors of HPV vaccination decision-making among parents of boys. Vaccine. 2017;35:4713–21. 10.1016/j.vaccine.2017.07.043. 28757059 10.1016/j.vaccine.2017.07.043

[108] Cunningham MS, Davison C, Aronson KJ. HPV vaccine acceptability in Africa: a systematic review. Prev Med. 2014;69:274–9. 10.1016/j.ypmed.2014.08.035. 25451327 10.1016/j.ypmed.2014.08.035

[109] Dochez C, Burnett RJ, Mbassi SM, Were F, Musyoki A, Trovoada D, Mphahlele MJ. Improving skills and institutional capacity to strengthen adolescent immunisation programmes and health systems in African countries through HPV vaccine introduction. Papillomavirus Res. 2017;4:66–71. 10.1016/j.pvr.2017.08.003. 29179872 10.1016/j.pvr.2017.08.003 PMC7268102

[110] Victory M, Do TQN, Kuo Y-F, Rodriguez AM. Parental knowledge gaps and barriers for children receiving human papillomavirus vaccine in the Rio Grande Valley of Texas. Hum Vaccin Immunother. 2019;15:1678–87. 10.1080/21645515.2019.1628551. 31170031 10.1080/21645515.2019.1628551 PMC6746477

[111] Dempsey AF, Butchart A, Singer D, Clark S, Davis M. Factors associated with parental intentions for male human papillomavirus vaccination: results of a national survey. Sex Transm Dis. 2011;38:769–76. 10.1097/OLQ.0b013e318211c248. 21336230 10.1097/OLQ.0b013e318211c248

[112] Sonawane K, Zhu Y, Montealegre JR, Lairson DR, Bauer C, McGee LU, et al. Parental intent to initiate and complete the human papillomavirus vaccine series in the USA: a nationwide, cross-sectional survey. Lancet Public Health. 2020;5:e484–92. 10.1016/S2468-2667(20)30139-0. 32707126 10.1016/S2468-2667(20)30139-0 PMC7484349

[113] Schuler CL, Coyne-Beasley T. Has their Son been Vaccinated? Beliefs about other parents Matter for Human Papillomavirus Vaccine. Am J Mens Health. 2016;10:318–24. 10.1177/1557988314567324. 25595021 10.1177/1557988314567324

[114] Kornides M, Head KJ, Feemster K, Zimet GD, Panozzo CA. Associations between HPV vaccination among women and their 11-14-year-old children. Hum Vaccin Immunother. 2019;15:1824–30. 10.1080/21645515.2019.1625642. 31295048 10.1080/21645515.2019.1625642 PMC6746495

[115] Bruni L, Diaz M, Barrionuevo-Rosas L, Herrero R, Bray F, Bosch FX, et al. Global estimates of human papillomavirus vaccination coverage by region and income level: a pooled analysis. Lancet Global Health. 2016;4:e453–63. 10.1016/S2214-109X(16)30099-7. 27340003 10.1016/S2214-109X(16)30099-7

[116] Solís Arce JS, Warren SS, Meriggi NF, Scacco A, McMurry N, Voors M, et al. COVID-19 vaccine acceptance and hesitancy in low- and middle-income countries. Nat Med. 2021;27:1385–94. 10.1038/s41591-021-01454-y. 34272499 10.1038/s41591-021-01454-y PMC8363502

[117] Aw J, Seng JJB, Seah SSY, Low LL. COVID-19 vaccine Hesitancy-A Scoping Review of Literature in High-Income Countries. Vaccines (Basel). 2021. 10.3390/vaccines9080900. 34452026 10.3390/vaccines9080900 PMC8402587

[118] Crow JM. HPV: the global burden. Nature. 2012;488:S2–3. 10.1038/488S2a. 22932437 10.1038/488S2a

[119] de Sanjosé S, Diaz M, Castellsagué X, Clifford G, Bruni L, Muñoz N, Bosch FX. Worldwide prevalence and genotype distribution of cervical human papillomavirus DNA in women with normal cytology: a meta-analysis. Lancet Infect Dis. 2007;7:453–9. 10.1016/S1473-3099(07)70158-5. 17597569 10.1016/S1473-3099(07)70158-5

[120] ICO-IARC HPV Information Centre. Statistics. https://hpvcentre.net/datastatistics.php. Accessed 14 Dec 2022.

[121] Marlow LAV, Wardle J, Forster AS, Waller J. Ethnic differences in human papillomavirus awareness and vaccine acceptability. J Epidemiol Community Health. 2009;63:1010–5. 10.1136/jech.2008.085886. 19762455 10.1136/jech.2008.085886 PMC3960938

[122] Tozzi AE, Ravà L, Stat D, Pandolfi E, Marino MG, Ugazio AG. Attitudes towards HPV immunization of Italian mothers of adolescent girls and potential role of health professionals in the immunization program. Vaccine. 2009;27:2625–9. 10.1016/j.vaccine.2009.02.050. 19428869 10.1016/j.vaccine.2009.02.050

[123] Ilter E, Celik A, Haliloglu B, Unlugedik E, Midi A, Gunduz T, Ozekici U. Women’s knowledge of pap smear test and human papillomavirus: acceptance of HPV vaccination to themselves and their daughters in an islamic society. Int J Gynecol Cancer. 2010;20:1058–62. 10.1111/IGC.0b013e3181dda2b9. 20683417 10.1111/IGC.0b013e3181dda2b9

[124] Morison LA, Cozzolino PJ, Orbell S. Temporal perspective and parental intention to accept the human papillomavirus vaccination for their daughter. Br J Health Psychol. 2010;15:151–65. 10.1348/135910709X437092. 19402950 10.1348/135910709X437092

[125] Wu JP, Porch E, McWeeney M, Ohman-Strickland P, Levine JP. Knowledge and concerns related to the human papillomavirus vaccine among underserved Latina women. J Low Genit Tract Dis. 2010;14:155–61. 10.1097/LGT.0b013e3181d4e747. 20592548 10.1097/LGT.0b013e3181d4e747

[126] Yeganeh N, Curtis D, Kuo A. Factors influencing HPV vaccination status in a latino population; and parental attitudes towards vaccine mandates. Vaccine. 2010;28:4186–91. 10.1016/j.vaccine.2010.04.010. 20417261 10.1016/j.vaccine.2010.04.010

[127] Ben Natan M, Aharon O, Palickshvili S, Gurman V. Attitude of Israeli mothers with vaccination of their daughters against human papilloma virus. J Pediatr Nurs. 2011;26:70–7. 10.1016/j.pedn.2009.07.006. 21256414 10.1016/j.pedn.2009.07.006

[128] Cook EE, Venkataramani AS, Kim JJ, Tamimi RM, Holmes MD. Legislation to Increase Uptake of HPV Vaccination and Adolescent Sexual Behaviors. Pediatrics. 2018. 10.1542/peds.2018-0458 10.1542/peds.2018-0458 PMC6317562 30104422

[129] Brouwer AF, Delinger RL, Eisenberg MC, Campredon LP, Walline HM, Carey TE, Meza R. HPV vaccination has not increased sexual activity or accelerated sexual debut in a college-aged cohort of men and women. BMC Public Health. 2019;19:821. 10.1186/s12889-019-7134-1. 31238911 10.1186/s12889-019-7134-1 PMC6593582

[130] National Cancer Institute (NIH). Despite Proven Safety of HPV Vaccines, More Parents Have Concerns. https://www.cancer.gov/news-events/cancer-currents-blog/2021/hpv-vaccine-parents-safety-concerns. Accessed 31 Oct 2022.

[131] Newman PA, Logie CH, Doukas N, Asakura K. HPV vaccine acceptability among men: a systematic review and meta-analysis. Sex Transm Infect. 2013;89:568–74. 10.1136/sextrans-2012-050980. 23828943 10.1136/sextrans-2012-050980 PMC3812849

[132] Galbraith KV, Lechuga J, Jenerette CM, Moore LAD, Palmer MH, Hamilton JB. Parental acceptance and uptake of the HPV vaccine among african-americans and latinos in the United States: a literature review. Soc Sci Med. 2016;159:116–26. 10.1016/j.socscimed.2016.04.028. 27180256 10.1016/j.socscimed.2016.04.028 PMC12854015

[133] Gray A, Fisher CB. Factors associated with HPV vaccine acceptability and hesitancy among black mothers with young daughters in the United States. Front Public Health. 2023;11:1124206. 10.3389/fpubh.2023.1124206. 37139381 10.3389/fpubh.2023.1124206 PMC10150885

[134] Washington A, Chabaan J, Fakih A, Ford S, Rutledge L, Lilly J, et al. Why is it so necessary? African American parents’ perspectives on delaying and refusing HPV vaccination. J Pediatr Health Care. 2023;37:373–80. 10.1016/j.pedhc.2023.01.002. 36764888 10.1016/j.pedhc.2023.01.002

[135] Garcia S, Hopfer S, Amaro H, Tanjasiri S. HPV vaccine delay and refusal among unvaccinated Mexican American young adult women: a qualitative investigation of Mexican-born and US-born HPV vaccine decision narratives. J Behav Med. 2023;46:88–99. 10.1007/s10865-022-00326-1. 35610490 10.1007/s10865-022-00326-1 PMC9130004

[136] Glenn BA, Tsui J, Coronado GD, Fernandez ME, Savas LS, Taylor VM, Bastani R. Understanding HPV vaccination among latino adolescent girls in three U.S. regions. J Immigr Minor Health. 2015;17:96–103. 10.1007/s10903-014-9996-8. 24557745 10.1007/s10903-014-9996-8

[137] Chapman E, Venkat P, Ko E, Orezzoli JP, Del Carmen M, Garner EIO. Use of multimedia as an educational tool to improve human papillomavirus vaccine acceptability–a pilot study. Gynecol Oncol. 2010;118:103–7. 10.1016/j.ygyno.2010.04.010. 20457469 10.1016/j.ygyno.2010.04.010

[138] Francis SA, Nelson J, Liverpool J, Soogun S, Mofammere N, Thorpe RJ. Examining attitudes and knowledge about HPV and cervical cancer risk among female clinic attendees in Johannesburg, South Africa. Vaccine. 2010;28:8026–32. 10.1016/j.vaccine.2010.08.090. 20887829 10.1016/j.vaccine.2010.08.090

[139] Gillespie L, Hicks CW, Santana M, Worley SE, Banas DA, Holmes S, Rome ES. The acceptability of human papillomavirus vaccine among parents and guardians of newborn to 10-year-old children. J Pediatr Adolesc Gynecol. 2011;24:66–70. 10.1016/j.jpag.2010.07.004. 20709581 10.1016/j.jpag.2010.07.004

[140] Rand CM, Schaffer SJ, Humiston SG, Albertin CS, Shone LP, Heintz EV, et al. Patient-provider communication and human papillomavirus vaccine acceptance. Clin Pediatr (Phila). 2011;50:106–13. 10.1177/0009922810379907. 20837607 10.1177/0009922810379907

[141] Berenson AB, Brown VG, Fuchs EL, Hirth JM, Chang M. Relationship between maternal experiences and adolescent HPV vaccination. Hum Vaccin Immunother. 2017;13:2150–4. 10.1080/21645515.2017.1332551. 28604258 10.1080/21645515.2017.1332551 PMC5612520

[142] Baumann A, Andersen B, Østergaard L, Larsen MB. Sense & sensibility: decision-making and sources of information in mothers who decline HPV vaccination of their adolescent daughters. Vaccine X. 2019;2:100020. 10.1016/j.jvacx.2019.100020. 31384740 10.1016/j.jvacx.2019.100020 PMC6668232

[143] Keelan J, Pavri V, Balakrishnan R, Wilson K. An analysis of the human papilloma virus vaccine debate on MySpace blogs. Vaccine. 2010;28:1535–40. 10.1016/j.vaccine.2009.11.060. 20003922 10.1016/j.vaccine.2009.11.060

[144] Kahn JA, Ding L, Huang B, Zimet GD, Rosenthal SL, Frazier AL. Mothers’ intention for their daughters and themselves to receive the human papillomavirus vaccine: a national study of nurses. Pediatrics. 2009;123:1439–45. 10.1542/peds.2008-1536. 19482752 10.1542/peds.2008-1536

[145] Brewer NT, Chapman GB, Rothman AJ, Leask J, Kempe A. Increasing vaccination: putting Psychological Science Into Action. Psychol Sci Public Interest. 2017;18:149–207. 10.1177/1529100618760521. 29611455 10.1177/1529100618760521

[146] Ogilvie GS, Remple VP, Marra F, McNeil SA, Naus M, Pielak KL, et al. Parental intention to have daughters receive the human papillomavirus vaccine. CMAJ. 2007;177:1506–12. 10.1503/cmaj.071022. 18056599 10.1503/cmaj.071022 PMC2096481

[147] Podolsky R, Cremer M, Atrio J, Hochman T, Arslan AA. HPV vaccine acceptability by latino parents: a comparison of U.S. and Salvadoran populations. J Pediatr Adolesc Gynecol. 2009;22:205–15. 10.1016/j.jpag.2008.05.010. 19646665 10.1016/j.jpag.2008.05.010

[148] Karafillakis E, Simas C, Jarrett C, Verger P, Peretti-Watel P, Dib F, et al. HPV vaccination in a context of public mistrust and uncertainty: a systematic literature review of determinants of HPV vaccine hesitancy in Europe. Hum Vaccin Immunother. 2019;15:1615–27. 10.1080/21645515.2018.1564436. 30633623 10.1080/21645515.2018.1564436 PMC6783136

[149] Hendry M, Lewis R, Clements A, Damery S, Wilkinson C. HPV? Never heard of it! A systematic review of girls’ and parents’ information needs, views and preferences about human papillomavirus vaccination. Vaccine. 2013;31:5152–67. 10.1016/j.vaccine.2013.08.091. 24029117 10.1016/j.vaccine.2013.08.091

[150] Bratic JS, Seyferth ER, Bocchini JA. Update on barriers to human papillomavirus vaccination and effective strategies to promote vaccine acceptance. Curr Opin Pediatr. 2016;28:407–12. 10.1097/MOP.0000000000000353. 27093354 10.1097/MOP.0000000000000353

[151] Wong CA, Berkowitz Z, Dorell CG, Price RA, Lee J, Saraiya M. Human papillomavirus vaccine Uptake among 9–17 Year Old girls National Health interview Survey, 2008. Cancer. 2011;117:5612–20. 10.1002/cncr.26246. 21692069 10.1002/cncr.26246 PMC3179804

[152] Al-Naggar RA, Bobryshev YV, Al-Jashamy K, Al-Musli M. Practice of HPV vaccine and associated factors among school girls in Melaka, Malaysia. Asian Pac J Cancer Prev. 2012;13:3835–40. 10.7314/apjcp.2012.13.8.3835. 23098480 10.7314/apjcp.2012.13.8.3835

[153] Li SL, Lau YL, Lam TH, Yip PSF, Fan SYS, Ip P. HPV vaccination in Hong Kong: uptake and reasons for non-vaccination amongst Chinese adolescent girls. Vaccine. 2013;31:5785–8. 10.1016/j.vaccine.2013.10.027. 24148571 10.1016/j.vaccine.2013.10.027

[154] Reiter PL, McRee A-L, Pepper JK, Gilkey MB, Galbraith KV, Brewer NT. Longitudinal predictors of human papillomavirus vaccination among a national sample of adolescent males. Am J Public Health. 2013;103:1419–27. 10.2105/AJPH.2012.301189. 23763402 10.2105/AJPH.2012.301189 PMC3725571

[155] Feiring B, Laake I, Molden T, Cappelen I, Håberg SE, Magnus P, et al. Do parental education and income matter? A nationwide register-based study on HPV vaccine uptake in the school-based immunisation programme in Norway. BMJ Open. 2015;5:e006422. 10.1136/bmjopen-2014-006422. 25991445 10.1136/bmjopen-2014-006422 PMC4442157

[156] Hansen BT, Campbell S, Burger E, Nygård M. Correlates of HPV vaccine uptake in school-based routine vaccination of preadolescent girls in Norway: a register-based study of 90,000 girls and their parents. Prev Med. 2015;77:4–10. 10.1016/j.ypmed.2015.04.024. 25944266 10.1016/j.ypmed.2015.04.024

