# Supplementary material for: Global parental acceptance, attitudes, and knowledge regarding human papillomavirus vaccinations for their children: a systematic literature review and meta-analysis
Source: BMC Womens Health. 2024 Sep 27;24:537. doi: 10.1186/s12905-024-03377-5 (PMC11428909; doi:10.1186/s12905-024-03377-5)
Supplement: Supplementary file 1 — Supplementary Material 1 [file 12905_2024_3377_MOESM1_ESM.docx]

**Additional data–Appendix A**

**Additional file 1:** Overview of the complete search strategy

**Databases:** PubMed, Web of Science and Scopus

**Search date:** 09 October 2023

**Search period:** 1 January 2006 to 09 October 2023

**Search filter:**  Publication date from 1 January 2006 to 31 December 2023

Species Humans

Language Englisch and German

**Search strategy (PubMed)**

| **Set #** | **MeSH/tiab terms** | **Results** |
| --- | --- | --- |
| 1 | "Parents"[Mesh] OR "parent*"[tiab] OR "caregiver*"[tiab] OR parental[tiab] OR mother*[tiab] OR father*[tiab] OR "Child"[Mesh] OR "children"[tiab] OR daughter*[tiab] OR son[tiab] | 1,244,222 |
| 2 | "health knowledge"[tiab] OR "health information"[tiab] OR information[tiab] OR inform*[tiab] OR knowledge*[tiab] OR attitude*[tiab] OR awareness*[tiab] OR perception[tiab] OR perceive*[tiab] OR refusal[tiab] OR refuse*[tiab] OR acceptance[tiab] OR accept*[tiab] OR willingness[tiab] OR willing*[tiab] OR intention*[tiab] | 1,760,799 |
| 3 | "Papillomavirus Infections"[Mesh] OR "human papilloma virus"[tiab] OR "human papillomavirus"[tiab] OR "papillomavirus"[All Fields] OR "HPV"[All Fields] OR "sexually transmitted infection"[All Fields] OR "STI" [All Fields] OR "sexually transmitted disease"[All Fields] OR "STD"[All Fields] | 66,201 |
| 4 | "Papillomavirus Vaccines"[Mesh] OR "Vaccines"[Mesh] OR "Vaccination"[Mesh] OR "Immunization"[Mesh] OR "Immunization Schedule"[Mesh] OR vaccine*[tiab] OR vaccination[tiab] OR vaccinate*[tiab] OR immunization*[tiab] OR immunisation*[tiab] OR HPV[tiab] OR "HPV vaccination*"[tiab] OR "HPV vaccine*"[tiab] OR "HPV immunization*"[tiab] OR "human papilloma virus vaccine*"[tiab] OR "human papilloma virus vaccination"[tiab] OR "human papilloma virus immunization*"[tiab] OR "HPV prevention*" OR "human papilloma virus prevention*" | 228,724 |
| 5 | systematic review[tiab] OR systematic literature review[tiab] OR literature review[tiab] OR narrative review[tiab] OR scoping review[tiab] | 302,577 |
| 6 | **#1 AND #2 AND #3 AND #4 NOT #5** | **2,063** |

**Search strategy (Web of Science)**

| **Set #** | **MeSH/tiab terms** | **Results** |
| --- | --- | --- |
| 1 | TS=(Parents OR Child) OR TI=(parent* OR caregiver* OR parental OR mother*OR father*OR children OR daughter* OR son) OR AB=( parent* OR caregiver* OR parental OR mother*OR father*OR children* OR daughter* OR son) | 1,722,294 |
| 2 | TI=(health knowledge OR health information OR information OR inform* OR knowledge* OR attitude* OR awareness*OR perception OR perceive* OR refusal OR refuse* OR acceptance OR accept* OR willingness OR willing* OR intention*) OR AB=(health knowledge OR health information OR information OR inform* OR knowledge* OR attitude* OR awareness*OR perception OR perceive* OR refusal OR refuse* OR acceptance OR accept* OR willingness OR willing* OR intention*) | 6,558,540 |
| 3 | TS=(Papillomavirus Infections OR papillomavirus OR HPV OR sexually transmitted infection OR STI OR sexually transmitted disease OR STD) OR TI=(human papilloma virus OR human papillomavirus) OR AB=(human papilloma virus OR human papillomavirus) | 132,288 |
| 4 | TS=(Papillomavirus Vaccines OR Vaccines OR Vaccination OR Immunization OR Immunization Schedule) OR TI=(vaccine* OR vaccination OR vaccinate* OR immunization* OR immunisation* OR HPV OR HPV vaccination* OR HPV vaccine* OR HPV immunization* OR "human papilloma virus vaccine* OR human papilloma virus vaccination OR human papilloma virus immunization* OR HPV prevention* OR human papilloma virus prevention*) OR AB=(vaccine* OR vaccination OR vaccinate* OR immunization* OR immunisation* OR HPV OR HPV vaccination* OR HPV vaccine* OR HPV immunization* OR "human papilloma virus vaccine* OR human papilloma virus vaccination OR human papilloma virus immunization* OR HPV prevention* OR human papilloma virus prevention*) | 488,019 |
| 5 | TI=(systematic review OR systematic literature review OR literature review[tiab] OR narrative review OR scoping review) | 292,451 |
| 6 | **#1 AND #2 AND #3 AND #4 NOT #5** | **1,884** |

**Search strategy (Scopus)**

| **Set #** | **Terms** | **Results** |
| --- | --- | --- |
| 1 | ALL (parents OR parent* OR caregiver* OR parental OR mother* OR father*OR child OR children OR daughter* OR son) | 1,604,896 |
| 2 | ALL (health AND knowledge OR health AND information OR information OR inform* OR knowledge OR attitude* OR awareness* OR perception OR perceive* OR refusal OR refuse* OR acceptance OR accept* OR willingness OR willing* OR intention*) | 6,122,590 |
| 3 | ALL (papillomavirus AND infections OR human AND papilloma AND virus OR human AND papillomavirus OR papillomavirus OR HPV OR sexually AND transmitted AND infection OR STI OR sexually AND transmitted AND disease OR STD) | 7,024 |
| 4 | ALL (papillomavirus AND vaccines OR vaccines OR vaccination OR immunization OR immunization AND schedule OR vaccine* OR vaccination OR vaccinate* OR immunization* OR immunisation* OR HPV OR HPV AND vaccination OR HPV AND vaccine* OR HPV AND immunization* OR human AND papilloma AND virus AND vaccine* OR human AND papilloma AND virus AND vaccination OR human AND papilloma AND virus AND immunization OR HPV AND prevention OR human AND papilloma AND virus AND prevention*) | 11,684 |
| 5 | ALL (systematic review OR systematic literature review OR literature review OR narrative review OR scoping review) | 1,982,915 |
| 6 | **#1 AND #2 AND #3 AND #4 NOT #5** | **668** |

**Additional file 2:** Newcastle-Ottawa Scale for cohort studies

**Note:** A study can be awarded a maximum of one star for each numbered item within the Outcome categories. A maximum of two stars can be given for Comparability.

**Selection (Maximum 3 stars):**

1. **Representativeness of the cohort**
2. truly representative of the average in the community ★
3. somewhat representative of the average in the community ★
4. selected group of users
5. no description of the derivation of the cohort
6. **Ascertainment of the exposure**
7. secure record (e.g. medical records) ★
8. structured interview ★
9. written self report
10. no description
11. **Demonstration that outcome of interest was not present at start of study**
12. yes ★
13. no

**Comparability (Maximum 2 stars):**

1. **The subjects in different cohorts (e.g. intenders and non-intenders; "undecided" or "hesitant" group) are comparable, based on the study design or analysis. Confounding factors are controlled.**
2. the study controls for the most important factor ★
3. the study control for any additional factor ★
4. data/results not adjusted for all relevant confounders/risk; factors/information not provided

**Outcome (Maximum 3 stars):**

1. **Assessment of outcome**
2. independent blind assessment ★
3. record linkage ★
4. self-report
5. no description
6. **The follow-up was long enough to occur the outcomes**
7. yes ★
8. no
9. **Adequacy of follow up of cohorts**
10. complete follow up (all subjects accounted for) ★
11. subjects lost to follow up unlikely to introduce bias, description provided of those lost ★
12. no description of those lost
13. no statement

**Rating:**

Very Good: 7 to 9 stars

Good: 5 to 6 stars

Satisfactory: 3 to 4 stars

Unsatisfactory: 0 to 2 stars

**Additional file 3:** Newcastle-Ottawa Scale adapted for cross-sectional studies

**Note:** This scale has been adapted from the Newcastle-Ottawa Scale for cohort studies to provide quality assessment of cross-sectional studies. A study can be awarded a maximum of one star for each numbered item within the Selection and Outcome categories. A maximum of two stars can be given for Comparability.

**Selection (Maximum 5 stars):**

1. **Representativeness of the sample**
2. truly representative of the average in the target population **★**

(all subjects or random sampling)

1. somewhat representative of the average in the target population **★**

(non-random sampling)

1. selected group of users
2. no description of the sampling strategy
3. **Sample size**
4. justified and satisfactory (including sample size calculation) ★
5. not justified
6. no information provided
7. **Non-respondents**
8. comparability between respondents and non-respondents’ characteristics is established, and the response rate is satisfactory ★
9. the response rate is unsatisfactory, or the comparability between respondents and non-respondents is unsatisfactory
10. no description of the response rate or the characteristics of the responders and the non-responders
11. **Ascertainment of the exposure (risk factor)**
12. validated measurement tool ★★
13. non-validated measurement tool, but the tool is available or described ★
14. no description of the measurement tool

**Comparability (Maximum 2 stars):**

1. **The subjects in different outcome groups are comparable, based on the study**

**design or analysis. Confounding factors are controlled.**

1. the study controls for the most important factor (select one) ★
2. the study control for any additional factor ★
3. data/results not adjusted for all relevant confounders/risk; factors/information not provided

**Outcome (Maximum 3 stars):**

1. **Ascertainment of the exposure (risk factor)**
2. independent blind assessment ★
3. record linkage ★
4. self-report
5. no description
6. **Statistical test**
7. the statistical test used to analyse the data is clearly described and appropriate, and the measurement of the association is presented, including confidence intervals and the probability level (p value) ★
8. the statistical test is not appropriate, not described or incomplete

**Rating:**

Very Good: 9 to 10 stars

Good: 7 to 8 stars

Satisfactory: 5 to 6 stars

Unsatisfactory: 0 to 4 stars

**Additional file 4:** Overall and stratified pooled vaccine acceptance by the investigated variables including the prediction intervals, publication bias and quality assessment

| **Vaccine acceptance** | **Strata** | **Number of studies** | **Pooled vaccine acceptance (95% CI)** | **Predicition interval of vaccine acceptance (%)** | **Publication bias** | **Quality of evidence (GRADE)^a^** |
| --- | --- | --- | --- | --- | --- | --- |
| **Overall** |  | **62** | **67.2% (95% CI: 62.6–71.7)** | **30.1–94.8** | **Undetected** | ⨁⨁⨁⨀ moderate |
| **Subgroup analysis** | | | | | | |
| **Stratified by** | Africa | 17 | 79.6% (95% CI: 73.5–85.2) | 51.1–97.4 | Undetected | ⨁⨁⨁⨀ moderate |
|  | Asia | 13 | 63.7% (95% CI: 55.4–71.6) | 32.8–89.4 | Undetected | ⨁⨀⨁⨀ low |
|  | Europe | 11 | 65.9% (95% CI: 51.7–78.8) | 18.8–98.5 | Undetected | ⨀⨀⨁⨀ very low |
|  | North America | 18 | 56.7% (95% CI: 49.3–64.0) | 25.8–85.0 | Undetected | ⨁⨀⨁⨀ low |
| **Sensitivity analyses** | | | | | | |
| **Stratified by** | Parents and children | 17 | 63.3% (95% CI: 52.9–73.1) | 21.0–95.8 | Undetected | ⨁⨀⨁⨀ low |
|  | Parents and daughters | 22 | 70.3% (95% CI: 62.9–77.1) | 34.1–95.8 | Undetected | ⨁⨀⨁⨀ low |
|  | Parents and sons | 10 | 57.7% (95% CI: 4.77–67.5) | 25.7–86.5 | Undetected | ⨀⨀⨁⨀ very low |
|  | Mothers and daughters | 13 | 73.9% (95% CI: 65.7–81.3) | 41.8–95.9 | Undetected | ⨁⨀⨁⨀ low |
|  | Parents and children + Asia | 7 | 58.4% (95% CI: 45.4–70.8) | 23.4–89.1 | Undetected | ⨀⨀⨁⨀ very low |
|  | Parents and children + Asia | 7 | 58.4% (95% CI: 45.4–70.8) | 23.4–89.1 | Undetected | ⨀⨀⨁⨀ very low |
|  | Parents and children + North America | 3 | 52.0% (95% CI: 16.6–86.3) | 0.1–100.0 | Undetected | ⨁⨀⨁⨀ low |
|  | Parents and daughters + Africa | 9 | 76.4% (95% CI: 66.3–85.3) | 40.7–98.2 | Undetected | ⨁⨀⨁⨀ low |
|  | Parents and daughters + Asia | 2 | 75.8% (95% CI: 67.1–83.6) | 60.7–88.2 | Undetected | ⨀⨁⨀⨀ very low |
|  | Parents and daughters + Europe | 2 | 66.3% (95% CI: 11.1–100) | 0.0–100.0 | Undetected | ⨀⨀⨀⨀ very low |
|  | Parents and daughters + North America | 7 | 60.7% (95% CI: 51.5–69.6) | 34.9–83.7 | Detected | ⨁⨀⨁⨀ low |
|  | Parents and sons + Europe | 3 | 63.9% (95% CI:38.1–86.0) | 15.7–98.4 | Detected | ⨀⨀⨀⨀ very low |
|  | Parents and sons + North America | 5 | 51.2% (95% CI:42.2–-60.2) | 29.9–72.3 | Undetected | ⨀⨁⨀⨀ very low |
|  | Mothers and daughters + Africa | 5 | 86.3% (95% CI: 81.6–90.5) | 75.5–94.9 | Undetected | ⨀⨁⨀⨀ very low |
|  | Mothers and daughters + Asia | 3 | 71.5% (95% CI: 66.2–76.5) | 60.9–81.0 | Undetected | ⨀⨀⨀⨀ very low |
|  | Mothers and daughters + North America | 3 | 61.2% (95% CI:48.7–73.0) | 36.7–83.0 | Detected | ⨀⨁⨀⨀ very low |

^a^ Limitation, inconsistency, indirectness, imprecision in the order listed in the table: ⨀—serious or ⨁—non-serious.

**Additional file 5**: Funnel plot of HPV vaccine acceptance rates (n = 62)


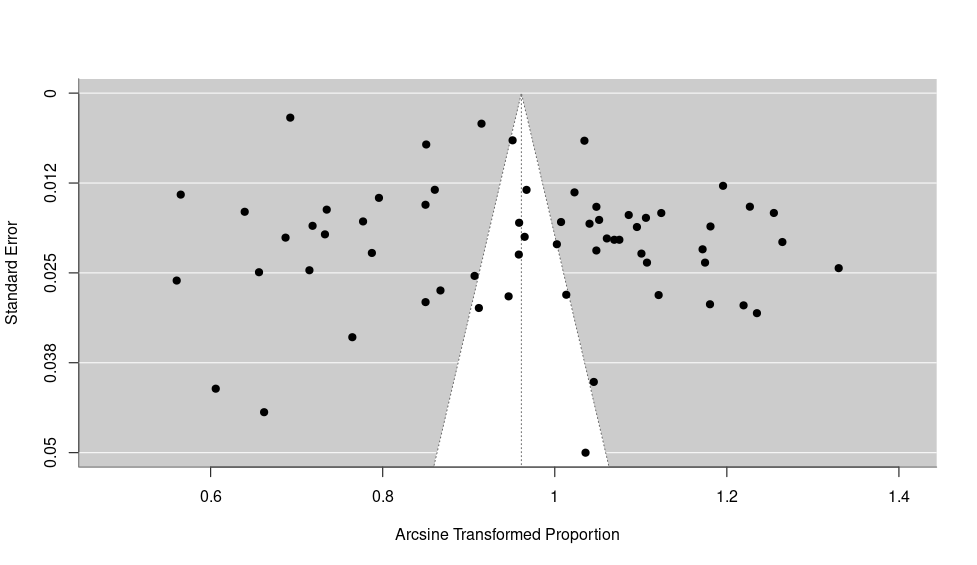


**Additional file 6:** Characteristics of the included studies and vaccine acceptance rate

| **Reference** | **Study**  **region** | **Study**  **period** | **Study setting** | **Parents sample size (n)** | **Parents’ age  (years)** | **Children’s age (years)** | **Parents’ sex** | **Children’s sex** | **Parents’ ethnicity** | **Survey**  **instrument** | **Acceptance rate (%)** | **Parents sample size (n) for**  **acceptance rate (%)** | **Intention vs. already vaccinated** | **Sampling**  **method** |
| --- | --- | --- | --- | --- | --- | --- | --- | --- | --- | --- | --- | --- | --- | --- |
| Brabin (2006) | Manchester, England | 2005 | Parents of year 7 (age 11–12) pupils in the city of Manchester, school data of Manchester City Council Education Department | 317 | ≤ 30 to > 45 | 11–12 | F/M | F/M | 65.3% White,  7.9% Black-Caribbean,  8.8% Black-African,  12.3% Indian, 4.1% Others | Paper-based questionnaire | 81.1 | 257 | Intention | Stratified sampling |
| Constantine (2007) | California, USA | 2005–2006 | Subset of data from survey on sexual health in California | 522 | < 30 to > 50 | ≤ 18 | F/M | F | 40.5% White, 38.2% Hispanic, 6.9% Black, 7.7% Asian, 5.4% Other | Interviewer-administered questionnaire  (telephone interview) | 75.1 | 392 | Intention | Stratified sampling |
| Hopenhayn (2007) | Appalachian Kentucky, USA | 2005 | Two counties in Appalachian Kentucky | 626 | 18 to > 70 | 10–15 | F | F | 95% White | Computer assisted telephone interview (CATI) | 67.6 | 423 | Intention | Simple  random sampling |
| Marshall (2007) | Not specified, Australia | 2006 | Households in South  Australia | 601 | 47 (mean) | N/A | F/M | F/M | N/A | Interviewer-administered questionnaire  (telephone interview) | 76.9 | 462 | Intention | Simple  random sampling |
| Woodhall (2007) | Tampere, Finland | 2005 | Survey-based study among adolescents born in 1990 and their parents, targeting households | 727 | 45 (mean) | 15 (mean) | F/M | F/M | N/A | Paper-based questionnaire | 85.6 | 622 | Intention | Stratified sampling |
| Ogilvie (2008) | Not specified, Canada | 2006–2007 | National survey in Canada | 1381 | 19 to > 60 | 8–18 | F/M | M | 83.4% White, 2.3% Aboriginal, 14.3% Other | Computer assisted telephone interview (CATI) | 67.8 | 936 | Intention | Simple  random sampling |
| Bernat (2009) | Minnesota, USA | 2006–2007 | Random sample of parents of school-age children in Minnesota | 1504 | ≤ 30 to ≥ 50 | 5–18 | F/M | F/M | 96.8% White,  3.2% Non-White,  1.9% Hispanic | Interviewer-administered questionnaire  (telephone interview) | 86.6 | 1302 | Intention | Stratified sampling |
| Gottlieb (2009) | North  Carolina, USA | 2007 | Five North Carolina counties with high cervical cancer incidence | 780 | ≤ 39 to ≥ 50 | 10–18 | F/M | F | 52% White, 38% Black, 5% Hispanic,  5% Other | Interviewer-administered questionnaire  (telephone interview) | 62.1 | 484 | Vaccinated | Clustered sampling |
| Reiter  (2009) | North  Carolina, USA | 2007 | Counties with high risk of cervical cancer | 886 | < 40 to > 40 | 10–18 | F/M | F | 70.2% White, 23.2% Black, 6.6% Other | Interviewer-administered questionnaire  (telephone interview) | 12.0 | 106 | Vaccinated | Clustered sampling |
| Allen  (2010) | Not specified, USA | 2007–2008 | National internet-based survey in the USA | 448 | N/A | 9–17 | F/M | F | 48% White,  30% Black,  22% Hispanic | Web-based questionnaire | 52.9 | 237 | Mixed | Simple random sampling |
| Askelson (2010) | Iowa, USA | 2007 | Rural, Midwest state in the USA | 217 | 27–56 | 9–15 11.21 ± 1.82 (mean) | F | F | N/A | Paper-based questionnaire | 47.9 | 104 | Intention | Simple random sampling |
| Dahlström (2010) | Stockholm, Sweden | 2007 | Data from Swedish Population Register | 13840 | < 41 to > 45 | 12–15 | F/M | F/M | 86.5% Sweden, 3.1% Other Nordic country, 6.1% Other country | Paper-based questionnaire and telephone interview | 62.8 | 8691 | Intention | Simple random sampling |
| Fang  (2010) | Not specified, USA | 2007 | Data from the 2007 Health Information National Trends Survey | 1383 | 44 (mean) | ≤ 18 | F/M | F | 58.9% White, 12.6% Black, 20.4% Hispanic, 8.1% Other | Paper-based questionnaire and telephone interview | 57.5 | 795 | Intention | Simple random sampling |
| Mortensen (2010) | Not specified, Denmark | 2010 | Four countries (UK, Germany, France and Italy) randomly extracted from Yellow Pages, omnibus and door to door | 450 | N/A | 12–15 | F/M | M | N/A | Computer assisted telephone interview (CATI) | 80.0 | 360 | Intention | Simple random sampling |
| Oh  (2010) | Not specified, Korea | 2007 | National population-based cross-sectional survey in Korea | 1000 | 20 to ≥ 50 | N/A | F/M | F/M | N/A | Interviewer-administered questionnaire  (face-to-face interview) | 75.1 | 751 | Intention | Stratified sampling |
| Reiter  (2010) | North Carolina, USA | N/A | North Carolina residents’ data from the BRFSS and CHAMP surveys | 617 | ≤ 39 to ≥ 50 | 10–17 13.6 ± 2.4 (mean) | F/M | F | N/A | Interviewer-administered questionnaire  (telephone interview) | 32.1 | 198 | Vaccinated | Simple random sampling |
| Dempsey (2011) | Michigan, USA | 2009 | Data from national, cross-sectional, web-based survey in the USA | 1178 | 18 to ≥ 60 | 0–17 | F/M | M | 65% White, 11% Black, 17% Hispanic, 7% Other | Web-based questionnaire | 51.0 | 601 | Intention | Simple random sampling |
| Guerry (2011) | Los Angeles, USA | 2008–2009 | Data from study to prevent pregnancy and sexually transmitted infections in middle and high school students | 387 | 22–71 | 11–18 14.5 ± 1.92 (mean) | F/M | F | 81% Hispanic, 16% Black | Interviewer-administered questionnaire  (telephone interview) | 62.0 | 240 | Intention | Clustered sampling |
| Kadis  (2011) | Not specified, USA | 2009 | National survey of mothers of adolescent females in the USA | 496 | 41.7 ± 7.4 (mean) | 11–14 | F | F | 54.5% White, 17.3% Black, 8.0% Hispanic | Web-based questionnaire | 66.9 | 332 | Intention | Simple random sampling |
| Litton  (2011) | Alabama, USA | 2008–2009 | National survey on female caregiver’s intention to vaccinate their daughters | 403 | < 40 to ≥ 50 | 11–14 | F | F/M | 89.8% White, 9.0% Black | Interviewer-administered questionnaire  (telephone interview) | 37.3 | 150 | Intention | Simple random sampling |
| M’Imunya (2011) | Nairobi,  Kenya | 2010 | Four primary schools within the Langata constituency in Nairobi County, Kenya focusing on girls in Standard five to eight and their parents/guardians | 332 | < 20.4 to > 50.14 | < 10.38 to > 15.3 | F/M | F | N/A | Self-administered questionnaire | 58.0 | 193^d^ | Intention | Simple random sampling |
| Arrossi (2012) | Buenos Aires,  Argentina | 2009–2010 | Population-based survey among women in Metropolitan Buenos Aires | 100 | 18–49 | 9–15 | F | F | N/A | Paper-based questionnaire and  face-to-face interview | 74.0 | 74 | Intention | Stratified sampling |
| Gefenaite (2012) | Not specified, Netherlands | 2009 | National survey of parents of girls born in 1996 targeted for HPV vaccination in the Netherlands | 469 | 35–55 | N/A | F | F | N/A | Paper-based questionnaire | 65.5 | 307 | Vaccinated | Simple random sampling |
| Gilkey  (2012) | North  Carolina, USA | 2010 | Data from population-based surveys (BRFSS and CHAMP) | 751 | 44 (mean) | 11–17  14.0 (mean) | F/M | F/M | N/A | Interviewer-administered questionnaire  (telephone interview) | 29.7 | 223 | Vaccinated | Simple random sampling |
| Pourat (2012) | California, USA | N/A | Subanalysis of females in 2007 California Health Interview Survey | 4896 | 18 to ≥ 56 | 8–17 | F/M | F | 38% White, 5% Black, 44% Latino, 11% Asian, 2% Other | Interviewer-administered questionnaire (telephone interview) | 56.5 | 2766 | Intention | Simple random sampling |
| Rose  (2012) | Not specified, New Zealand | 2008–2009 | Parents of students in Wellington, New Zealand | 769 | 20 to ≥ 55 | ≥ 8 | F/M | F | 16.4% Maori, 7.4% Pacific, 62.0% New Zealand European, 14.2% Other | Paper-based questionnaire | 67.0 | 515 | Intention | Stratified sampling |
| Sadigh (2012) | Not specified, USA | 2006–2008 | National Survey of Family Growth, recruited by random digit dialing, USA | 444 | 15–44 | 9–18 | F | F | N/A | Paper-based questionnaire | 56.3 | 250 | Mixed | Simple random sampling |
| Songthap (2012) | Bangkok, Thailand | 2009 | School-based cross-sectional study among students, parents, and teachers in Bangkok, Thailand | 648 | < 40 to ≥ 50 | 12–15 | F/M | F/M | N/A | Self-administered questionnaire | 44.8 | 290 | Intention | Simple random sampling |
| Ezat  (2013) | Kuala  Lumpur, Malaysia | 2012 | Tertiary referral center in Malaysia, specifically targeting mothers who attended the Obstetrics and Gynecology (O&G) clinics | 155 | 24 to >33 | N/A | F | F/M | 82.6% Malaysian, 11.6% Chinese, 4.5% Indian, 1.3% Others | Self-administered questionnaire | 74.8^a^ | 116^a^ | Intention | Clustered sampling |
| Ezenwa (2013) | Lagos,  Nigeria | 2012 | Community-based survey with multistage sampling in Lagos State, Nigeria | 290 | 24–62 | 10–19 | F | F | 10.5% Hausa, 27.7% Igbo, 38.4% Yoruba, 23.5% Other | Paper-based questionnaire | 85.5 | 248 | Intention | Simple random sampling |
| Lai  (2013) | Not specified, USA | 2009 | National survey with data from Health Information National Trends Survey, USA | 804 | 18 to ≥ 65  44.9 ± 2.53 (mean) | N/A | F/M | F | 73% White, 24% Non-White, 3% Unreported | Paper-based questionnaire | 75.4 | 606 | Intention | Simple random sampling |
| McRee (2013) | Not specified, USA | 2010 | National survey with data from the HPV Immunization in Sons (HIS) study, USA | 506 | 40.1 ± 6.9 (mean) | 11–17 | F/M | M | 67.2% White, 12.3% Black, 15.4% Hispanic, 5.1% Other | Web-based questionnaire | 50.2 | 254 | Intention | Simple random sampling |
| van Keulen (2013) | Not specified, Netherlands | 2009–2010 | National survey with random sample from Dutch vaccination register | 952 | < 45 to ≥ 45 | 13–16  13 ± 0.5 (mean) | F | F | N/A | Paper-based questionnaire | 45.0 | 428 | Intention | Stratified sampling |
| Bianco (2014) | Calabria, Italy | 2012 | Seven public secondary schools | 566 | 42.9 (mean) | 10–14 | F/M | M | N/A | Paper-based questionnaire | 71.0 | 402 | Intention | Clustered sampling |
| Kruiroongroj (2014) | Bangkok, Thailand | N/A | Cross-sectional survey with eight secondary schools in Bangkok | 758 | 43.47 ± 6.6 (mean) | 12–15 13.72 ± 2 1.3 (mean) | F | F | N/A | Paper-based questionnaire | 74.4 | 564 | Intention | Clustered sampling |
| Madhivanan (2014) | Mysore City, India | 2010 | Parents of children in one of 12 schools in Mysore, India | 778 | ≤ 35 to ≥ 46 | 11–15 | F/M | F | N/A | Paper-based questionnaire | 71.5 | 556 | Intention | Clustered sampling |
| Muhwezi (2014) | Ibanda, Nakasongola, Mbarara,  Luwero, Uganda | 2008–2009 | Cross-sectional study conducted in four districts of Uganda | 870 | 40.77 (mean) | 10–23 | F/M | M | N/A | Self-administered questionnaire | 78.3 | 681 | Intention | Simple random sampling |
| Taylor  (2014) | Not specified, USA | 2012 | National sample of parents who were part of the SSI national sample | 758 | 42.2 ± 11.5 (mean) | 11–17 13.8 ± 2.0 (mean) | F/M | M | 73.8% White, 10.0% Black, 9.8% Hispanic, 6.5% Other | Web-based questionnaire | 34.3 | 260 | Vaccinated | Simple random sampling |
| Vermandere (2014) | Eldoret,  Kenya | 2012 | Randomly selected primary schools in Kenya | 287 | 21–59 | 8–18 | F | F | N/A | Interviewer-administered questionnaire  (face-to-face interview) | 88.2 | 253 | Intention | Clustered sampling |
| Krawczyk (2015) | Quebec,  Canada | 2008–2009 | Cross-sectional survey of parents of girls in Quebec, Canada | 774 | 35 (median) | 8–18 | F | F | 88.5% White, 1.4% Black, 1.8% Arabic, 2.1% Aboriginal, 2.5% Other | Paper-based questionnaire | 88.2 | 683 | Vaccinated | Simple random sampling |
| La Vincente (2015) | Melanesia, Republic of Fiji | 2009–2010 | Parents of vaccine-eligible girls in primary schools in Fiji | 293 | 40 (median) | 9–10 | F/M | F | N/A | Interviewer-administered questionnaire  (telephone interview) | 78.5 | 230 | Vaccinated | Clustered sampling |
| Morhason-Bello (2015) | Mokola Ibadan,  Nigeria | 2012 | Data from HPV Vaccine and Cervical Cancer Prevention household Survey in Mokala, Nigeria | 1002 | < 25 to ≥ 40 | 9–12 | F/M | F | 70.6% Yoruba, 11.4% Hausa, 4.2% Igbo, 10.4% Others, 3.4% No response | Web-based questionnaire | 88.6 | 888 | Intention | Clustered sampling |
| Moss  (2015) | Not specified, USA | 2010 | Data from HPV Immunization in Sons survey, USA | 412 | < 45 to ≥ 45 | 11–17 | F/M | M | N/A | Web-based questionnaire | 43.0 | 177 | Intention | Simple random sampling |
| Selmouni (2015) | Not specified, Morocco | 2011 | National cross-sectional surveys of mothers and fathers of girls at middle school in Morocco | 1312 | 41.8 ± 6.9 (mean) | 12–15 | F/M | F | N/A | Interviewer-administered questionnaire  (face-to-face interview) | 72.9 | 956 | Intention | Clustered sampling |
| Wang L.D-L. (2015) | Hong Kong, China | 2014 | Survey of Chinese parents with at least one daughter in Hongkong | 368 | 49.3 ± 8.2 (mean) | 12–17 | F/M | F/M | 98.3% Han Chinese | Interviewer-administered questionnaire  (telephone interview) | 28.3 | 104 | Intention | Simple random sampling |
| Borena (2016) | Tyrol, Austria | 2015 | Parents of fourth grade school children from 20 primary schools in Tyrol – a region in west Austria. | 148 | 40.8 ± 5.7 (mean) | N/A | F/M | F/M | N/A | Self-administered questionnaire | 32.4 | 48 | Intention | Simple random sampling |
| Clark  (2016) | Michigan, USA | 2012 | National, cross-sectional survey of parents of children aged 9–17, USA | 734 | 24 to ≥ 45 | 11–17 | F/M | M | 60.74% White,  12.03% Black,  22.37% Hispanic,  4.87% Other | Web-based questionnaire | 43.3 | 318 | Intention | Simple random sampling |
| Dairo  (2016) | Ibadan,  Nigeria | N/A | Community-based study of parents of adolescents residing in the community in Ibadan North Local Government Area | 612 | 20 to > 50 37.3 ± 6.5 (mean) | 10–18 | F/M | F/M | 88% Yoruba, 9.3% Igbo, 1.1% Hausa, 1.6% Others | Interviewer-administered questionnaire | 76.1 | 466 | Intention | Systematic random sampling |
| Oddsson (2016) | Not specified, Iceland | 2008 | Parents or guardians of 12-year-old girls from a cohort of total 2,092 girls born in 1995 and registered in the Icelandic National Register | 583 | 30 to ≥ 60 | 12 | F/M | F | N/A | Self-administered questionnaire | 90.9 | 530 | Intention | Simple random sampling |
| Voidăzan (2016) | Tîrgu Mureş, Romania | 2014 | Parents of students grades 5 to 8 in Tîrgu Mureş, Romania | 918 | 28–57 | N/A | F/M | F | 66.0% Romanian,  33.3% Hungarian,  0.7% Roma | Paper-based questionnaire | 35.6 | 327 | Intention | Clustered sampling |
| Calo  (2017) | Not specified, USA | 2014–2015 | National survey of parents of adolescents, USA | 1255 | N/A | 11–17 14 (mean) | F/M | F/M | 71% White, 9% Black, 13% Hispanic,  7% Other | Web-based questionnaire | 28.7 | 360 | Intention | Simple random sampling |
| Cheruvu (2017) | Not specified, USA | 2008–2012 | Sub-sample of unvaccinated females from the National Immunization Survey-Teen (NIS-Teen) | 21467 | ≤34 to ≥ 45 | 13 –17 14.3 (mean) | F/M | F | 60.1% White 15.5% Black 16.8% Hispanic 7.6% Other | Paper-based questionnaire and telephone interview | 40.8 | 8751 | Intention | Simple random sampling |
| Gilkey  (2017) | Not specified, USA | 2014–2015 | National survey of parents of adolescents, USA | 1484 | N/A | 11–17 14 (mean) | F/M | F/M | 71% White, 9% Black, 13% Hispanic,  7% Other | Web-based questionnaire | 46.2 | 685 | Vaccinated | Simple random sampling |
| Ndejjo  (2017) | Bugiri and Mayuge, Uganda | N/A | Cross-sectional study on females aged 25 to 49 years in eastern Uganda | 900 | 25–49 | N/A | F | F | N/A | Paper-based questionnaire | 90.3 | 813 | Intention | Clustered sampling |
| Perez  (2017) | Not specified, Canada | 2014 | Nationally representative sample of Canadian parents of boys at baseline (February 2014: T1) and at follow-up (November 2014: T2) | 3011 (T1) 1336 (T2) | N/A | 9–16 | F/M | M | N/A | Web-based questionnaire | 5.0 (T1)  9.8 (T2) | 157 (T1) 140 (T2) | Mixed | Simple random sampling |
| VanWormer (2017) | Wisconsin, USA | 2015–2016 | Data from medical care quality improvement survey on HPV vaccine coverage in seven communities in Wisconsin, USA | 164 | 43.7 ± 6.1 (mean) | 11 to ≥ 14 | F/M | F/M | 96% White, 3% Hispanic, 1% Unknown | Paper-based questionnaire | 59.8 | 98 | Vaccinated | Stratified sampling |
| Degarege (2018) | Mysore, India | 2011 | Cross-sectional survey of parents of adolescent girls in Karnataka state, India | 831 | ≤ 35 to ≥ 50 37.1 ± 6.67 (mean) | 11–15 | F/M | F | N/A | Paper-based questionnaire | 79.9 | 664 | Intention | Clustered sampling |
| Ganczak (2018) | Zgorzelec, Poland | 2013–2014 | Cross-sectional study among parents of first-grade high school students from three high schools in the city of Zgorzelec, southwestern Poland | 450 | 29–67 42 (mean) | N/A | F/M | F/M | N/A | Self-administered questionnaire | 85.1 | 383 | Intention | Simple random sampling |
| Mansfield (2018) | Not specified, USA | 2006–2007 | Retrospective, cross-sectional study based on a national dataset, the Health Information National Trends Survey (HINTS) | 1037 | 18 to ≥ 48 | N/A | F/M | F | 68.9% White, 11.4% African American, 11.6% Hispanic, 8.1% Others | Interviewer-administered questionnaire | 56.4 | 585 | Intention | Simple random sampling |
| Mendes Lobão  (2018) | Belém, Belo Horizonte, Brasília, Porto Alegre, Rio de  Janeiro, São Paulo,  Salvador, Brazil | 2015–2016 | Random-digit-dial study of parents conducted in seven major Brazilian cities | 826 | 18–82 43.8 (mean) | 9–14 | F/M | F/M | 44% White, 41% Mixed, 14% Black, 1% Asian, 0.3% Indigenous​​ | Interviewer-administered questionnaire | 89.0^a^ | 735^a^ | Mixed | Simple random sampling |
| Mohd Sopian (2018) | Kota Bharu, Malaysia | 2015 | Cross-sectional study among parents of standard 5 at 10 primary schools in Malaysia | 280 | 42.5 ± 6.22 (mean) | 11 | F/M | F/M | 100% Malaysian | Paper-based questionnaire | 62.5 | 175 | Intention | Systematic random sampling |
| O'Leary (2018) | Denver, Colorado, USA | 2013 | Cross-sectional study among parents of girls selected from administrative data in a Denver, Colorado safety net system | 40 | N/A | 12–15 | F/M | F | 20.8% White, 57.5% Hispanic, 21.6% Others | Web-based questionnaire | 97.5 | 39 | Vaccinated | Simple random sampling |
| Shapiro (2018) | Not specified, Canada | 2016 | Nationally representative sample of Canadian parents | 3779 | 18–81 43.51 ± 6.86 (mean) | 9–16 | F/M | F/M | 85.3% White 14.8% Other | Web-based questionnaire | 46.3 | 1750 | Mixed | Simple random sampling |
| Wang Z. (2018) | China, Hong Kong | 2016 | Chinese parents of children aged 9–13 years in Hongkong, recruited by random calling | 296 | < 40 to ≥ 50 | 9–13 | F/M | F/M | N/A | Interviewer-administered questionnaire  (telephone interview) | 56.4 | 167 | Intention | Simple random sampling |
| Azuogu (2019) | Ebonyi, Nigeria | 2018 | Cross sectional study of mothers of female senior secondary school students in Ebonyi, Nigeria | 267 | 20 to ≥ 49 42 ± 8 (mean) | 1 to ≥ 29 | F | F | N/A | Paper-based questionnaire | 89.1 | 238 | Intention | Stratified sampling |
| Hirth  (2019) | Not specified, USA | 2008–2016 | Longitudinal study using data from the 2008–2018 National Immunization Survey-Teen (NIS-Teen) | 143721 | N/A | 13–17 14.9 (mean) | F/M | F/M | Hispanic | Interviewer-administered questionnaire  (telephone interview) | 36.8^b^ | 52855 | Intention | Stratified sampling |
| Lin  (2019) | Shenzhen, China | 2015 | Multistage, stratified cluster sampling from Cervical Cancer Prevention Network data in Shenzhen city, China | 5799 | < 30 to ≥ 40  37.09 ± 7.48 (mean) | N/A | F | F | 92.7% Han, 7.3% Other | Self-administered questionnaire | 66.3 | 3842 | Intention | Clustered sampling |
| Shibli  (2019) | Haifa, Israel | 2016–2017 | Cross-sectional study among mothers of 8th graders in Jewish and Arab schools in Haifa and Northern districts of Israel, during the school year | 313 | 42.9 ± 4.8 (mean) | 12–13 | F | F/M | N/A | Interviewer-administered questionnaire  (telephone interview) | 65.8 | 206 | Intention | Simple random sampling |
| Tatar  (2019) | Canada | 2016–2017 | Nationally representative sample of Canadian parents | 497 | 42.5 ± 6.9 (mean) | 9–16 | F/M | F | 83.6% White, 16.4% Other | Web-based questionnaire | 18.9 (Intention) 14.9 (Vaccinated) | 94  (Intention) 74 (Vaccinated) | Mixed | Simple random sampling |
| Alene  (2020) | Gondar town, Ethiopia | 2019 | Parents who had daughters living in Gondar town, specifically selected from both urban and rural kebeles | 899 | 21 to ≥ 40 39.9 ± 0.30 (mean) | 9 –17 | F/M | F | N/A | Interviewer-administered questionnaire | 81.3 | 731 | Intention | Simple random sampling |
| Della Polla (2020) | Naples and Salerno, Italy | 2019 | Parents with at least one child attending six randomly selected middle public schools | 435 | 29–63 44.3 ± 5.8 (mean) | 12 –13 | F/M | F/M | N/A | Paper-based questionnaire | 57.9 | 252 | Mixed | Simple random sampling |
| Huon  (2020) | Loire-Atlantique, France | 2017–2018 | Sample of middle and high school students in the Loire-Atlantique department, France | 127 | 44–48 | 13–18 | F/M | M | N/A | Paper-based questionnaire | 37.8 | 48 | Intention | Clustered sampling |
| Lin  (2020) | Shenzhen, China | 2015 | Representative females with children recruited from healthcare institutions through the Cervical Cancer Prevention Network | 5702 | 21–60  37.60 ± 7.36 (mean) | N/A | F | F | 92.7% Han, 7.3% Other | Self-administered questionnaire | 73.9 | 4213 | Intention | Stratified sampling |
| Rabiu  (2020) | Lagos State, Nigeria | N/A | Cross-sectional study focusing on two urban and two rural secondary schools | 318 | 30–59 | N/A | F/M | F | N/A | Paper-based questionnaire | 72.0 | 229 | Intention | Simple random sampling |
| Dereje (2021) | Addis Ababa, Ethiopia | 2021 | Community-based cross-sectional study conducted among parents or guardians with daughters Akaki Kality sub-city | 422 | 39.0 ± 9.9 (mean) | 9 –17 | F/M | F | N/A | Interviewer-administered questionnaire | 94.3 | 398 | Intention | Systematic random sampling |
| Destaw (2021) | Bench-Sheko zone, Ethiopia | 2020–2021 | Community-based cross-sectional study conducted among residents in the Bench Sheko zone, southwest Ethiopia | 502 | 28.6 ± 6.0 (mean) | 9–13 | F/M | F | N/A | Interviewer-administered questionnaire | 79.5 | 399 | Intention | Systematic random sampling |
| Humnesa (2022) | Not specified, Ethiopia​ | 2021 | Randomly selected kebele in Central Ethiopia, focusing on households with daughters aged 9 to 14 years | 619 | < 30 to ≥ 40 35.13 ± 7.69 (mean) | 9–14 | F/M | F | 91.4% Oromo, 8.6% Amhara | Interviewer-administered questionnaire | 40.2 | 249 | Intention | Simple random sampling |
| Larebo (2022) | Hossana, Ethiopia | 2021 | Community-based cross-sectional study conducted among parents and daughters in the Hadiya zone, southern Ethiopia | 530 | 18 to ≥ 40 38 ± 9.45 (mean) | 9–14 | F/M | F | 73.4% Hadiya,  9.4% Kembata,  1.5% Amhara, 2.1% Tigre, 1.9% Gurage, 1.7% Oromo, 3% Wolaita,  6% Gurage, 3% Silte | Interviewer-administered questionnaire | 84.9 | 450 | Intention | Stratified sampling |
| López  (2022) | Not specified, Spain | 2020 | Cross-sectional, multicenter study at twenty-four (public and private) pediatric offices in Spain | 1405 | ≤ 29 to ≥ 60 | 9–14 11.5 ± 1.6 (mean) | F/M | F/M | 97.4% Spanish, 2.6% Others | Paper-based questionnaire or web-based questionnaire | 86.0^c^ | 1209^c^ | Mixed | Stratified sampling |
| Mihretie (2022) | Debre Tabor, Ethiopia | 2015–2016 | Community-based cross-sectional study among parents of girls | 638 | 23–46 | 9–14 | F/M | F/M | N/A | Interviewer-administered questionnaire | 44.8 | 286 | Mixed | Simple random sampling |
| Nguyen (2022) | Huế, Vietnam | 2020 | Cross-sectional study conducted at secondary schools in Hue city, involving parents of male students | 785 | 29–62 42.9 ± 6.1 (mean) | 11–15 12.4 ± 1.2 (mean) | F/M | M | N/A | Self-administered questionnaire | 49.2 | 386 | Intention | Simple random sampling |
| Sinshaw (2022) | Debre Markos, Ethiopia | 2021 | Mothers who had eligible daughters living in Debre Markos town in selected kebeles | 601 | 39.4 ± 8.95 (mean) | 9–14 | F | F | N/A | Interviewer-administered questionnaire | 77.4 | 465 | Intention | Simple random sampling |
| Alaamri (2023) | Tabuk City, Saudi Arabia | 2022–2023 | Community-based study conducted in 65 intermediate schools in Tabuk City​ | 947 | 24–55 42.28 ± 6.82 (mean) | N/A | F/M | F | 91.7% Saudi, 8.3% Non-Saudi | Web-based questionnaire | 35.7 | 338 | Mixed | Simple random sampling |
| Aragaw (2023) | Debre Tabor, Ethiopia | 2021–2022 | Parents who had daughters living in Debre town, specifically selected from three kebeles | 721 | ≤ 29 to ≥ 40 40.88 ± 6.72 (mean) | 9 –14 | F/M | F | N/A | Interviewer-administered questionnaire  (face-to-face interview) | 79.1 | 570 | Intention | Simple random sampling |
| Lubeya (2023) | Lusaka, Zambia | 2021 | Cross-sectional study conducted in Lusaka district, enrolled participants from various social settings such as markets, saloons, and barber shops across six sub-districts and twelve zones | 400 | 44.3–47.1 (mean) | 15–18 | F/M | F | N/A | Interviewer-administered questionnaire | 53.8 | 215 | Vaccinated | Clustered sampling |
| Sobierajski (2023) | Not specified, Poland | 2022 | Representative of parents of children aged 9–15 in Poland | 360 | <30 to >49 | 9–15 | F/M | F/M | N/A | Interviewer-administered questionnaire  (telephone interview) | 59.2 | 213 | Mixed | Stratified sampling |

^a^ An average acceptance rate has been calculated to account for parents providing multiple responses for daughters and sons. The parent sample size (n) for the acceptance rate was calculated based on the average acceptance rate, due to missing data regarding separate responses for daughters and sons.

^b^ Based on the percentage data of non-intenders.

^c^ The acceptance rate was based on responses categorized as 'agree' and 'strongly agree'. The parent sample size (n) for the acceptance rate was calculated based on the average acceptance rate, due to missing data.

^d^ The parent sample size (n) for the acceptance rate was calculated based on the reported acceptance rate.

**Additional file 7:** Quality assessment results using the modified Newcastle-Ottawa Scale

| **Modified Newcastle-Ottawa Scale for cross-sectional studies** | | | | | | | | | |
| --- | --- | --- | --- | --- | --- | --- | --- | --- | --- |
|  | **Selection** | | | | **Comparability** | **Outcome** | |  |  |
| **Reference** | **Representativeness of the sample (Maximum:★)** | **Sample size (Maximum:★)** | **Non-respondents (Maximum:★)** | **Ascertainment of exposure  (Maximum:★★)** | **Comparability of subjects on the basis of design or analysis (Maximum:★★)** | **Assessment of outcome (Maximum:★★)** | **Statistical test (Maximum:★)** | **Total score**  **(out of 10)** | **Power** |
| Brabin (2006) | ★ | ★ |  | ★★ | ★★ | ★ | ★ | ★★★★★★★★(8) | Good |
| Constantine (2007) | ★ | ★ |  | ★★ |  | ★ | ★ | ★★★★★★(6) | Satisfactory |
| Hopenhayn (2007) | ★ | ★ |  | ★ | ★ | ★ | ★ | ★★★★★★ (6) | Satisfactory |
| Marshall (2007) | ★ |  | ★ | ★★ | ★ | ★ | ★ | ★★★★★★★★(7) | Good |
| Woodhall (2007) | ★ |  |  | ★ | ★ | ★ | ★ | ★★★★★(5) | Satisfactory |
| Ogilvie (2008) | ★ | ★ |  | ★★ | ★★ | ★ | ★ | ★★★★★★★★(8) | Good |
| Bernat (2009) | ★ |  |  | ★ | ★ | ★ | ★ | ★★★★★★★★(8) | Good |
| Gottlieb (2009) | ★ |  | ★ | ★ |  | ★ | ★ | ★★★★★(5) | Satisfactory |
| Reiter (2009) | ★ |  | ★ | ★ | ★★ | ★ | ★ | ★★★★★★★(7) | Good |
| Allen (2010) | ★ |  |  | ★★ | ★★ | ★ | ★ | ★★★★★★★(7) | Good |
| Askelson (2010) | ★ | ★ |  | ★★ |  | ★ | ★ | ★★★★★★ (6) | Satisfactory |
| Dahlström (2010) | ★ |  | ★ | ★ | ★ | ★ | ★ | ★★★★★★ (6) | Satisfactory |
| Fang (2010) | ★ |  | ★ | ★ | ★ | ★ | ★ | ★★★★★★ (6) | Satisfactory |
| Mortensen (2010) | ★ |  |  | ★ |  | ★ |  | ★★★(3) | Unsatisfactory |
| Oh (2010) | ★ |  |  | ★ | ★ | ★ | ★ | ★★★★★(5) | Satisfactory |
| Reiter (2010) | ★ |  |  | ★ | ★★ | ★ | ★ | ★★★★★★ (6) | Satisfactory |
| Dempsey (2011) | ★ | ★ |  | ★★ | ★★ | ★ | ★ | ★★★★★★★★(8) | Good |
| Guerry (2011) | ★ |  |  | ★ | ★★ | ★ | ★ | ★★★★★★(6) | Satisfactory |
| Kadis (2011) | ★ |  |  | ★ | ★ | ★ | ★ | ★★★★★(5) | Satisfactory |
| Litton (2011) | ★ | ★ |  | ★★ | ★ | ★ | ★ | ★★★★★★★(7) | Good |
| M’Imunya (2011) | ★ | ★ |  |  | ★ | ★ | ★ | ★★★★★(5) | Satisfactory |
| Arrossi (2012) | ★ |  |  | ★★ | ★ | ★ | ★ | ★★★★★★(6) | Satisfactory |
| Gefenaite (2012) | ★ |  |  | ★ | ★ | ★★ | ★ | ★★★★★★(6) | Satisfactory |
| Gilkey (2012) | ★ |  |  | ★ | ★★ | ★ | ★ | ★★★★★★(6) | Satisfactory |
| Pourat (2012) | ★ |  |  | ★ | ★ | ★ | ★ | ★★★★★(5) | Satisfactory |
| Rose (2012) | ★ |  |  | ★ | ★ | ★ | ★ | ★★★★★(5) | Satisfactory |
| Sadigh (2012) | ★ |  |  | ★★ | ★ | ★ | ★ | ★★★★★★ (6) | Satisfactory |
| Songthap (2012) | ★ |  |  | ★★ | ★ | ★ | ★ | ★★★★★★ (6) | Satisfactory |
| Ezat (2013) | ★ | ★ |  | ★★ | ★ | ★ | ★ | ★★★★★★★★(7) | Good |
| Ezenwa (2013) | ★ | ★ |  | ★ |  | ★ | ★ | ★★★★★(5) | Satisfactory |
| Lai (2013) | ★ |  |  | ★ | ★ | ★ | ★ | ★★★★★(5) | Satisfactory |
| McRee (2013) | ★ |  |  | ★★ | ★ | ★ | ★ | ★★★★★★ (6) | Satisfactory |
| van Keulen (2013) | ★ |  |  | ★★ | ★★ | ★★ | ★ | ★★★★★★★★(8) | Good |
| Bianco (2014) | ★ |  |  | ★★ | ★ | ★ | ★ | ★★★★★★ (6) | Satisfactory |
| Kruiroongroj (2014) | ★ | ★ | ★ | ★ |  | ★ | ★ | ★★★★★★ (6) | Satisfactory |
| Madhivanan (2014) | ★ |  | ★ | ★ | ★★ | ★ | ★ | ★★★★★★★(7) | Good |
| Muhwezi (2014) | ★ | ★ |  | ★★ |  | ★ | ★ | ★★★★★★(6) | Satisfactory |
| Taylor (2014) | ★ |  |  | ★ | ★ | ★ | ★ | ★★★★★(5) | Satisfactory |
| Vermandere (2014) | ★ | ★ |  | ★★ | ★ | ★ | ★ | ★★★★★★★(7) | Good |
| Krawczyk (2015) | ★ |  |  | ★★ | ★★ | ★ | ★ | ★★★★★★★(7) | Good |
| La Vincente (2015) | ★ |  | ★ | ★ | ★ | ★ | ★ | ★★★★★★(6) | Satisfactory |
| Morhason-Bello (2015) | ★ |  |  | ★★ | ★ | ★ | ★ | ★★★★★★(6) | Satisfactory |
| Moss (2015) | ★ |  | ★ | ★ | ★ | ★ | ★ | ★★★★★★(6) | Satisfactory |
| Selmouni (2015) | ★ |  | ★ | ★★ | ★ | ★ | ★ | ★★★★★★★(7) | Good |
| Wang L.D-L. (2015) | ★ |  |  | ★ | ★ | ★ | ★ | ★★★★★(5) | Satisfactory |
| Borena (2016) | ★ |  |  | ★ | ★ | ★ | ★ | ★★★★★(5) | Satisfactory |
| Clark (2016) | ★ |  |  | ★ |  | ★ | ★ | ★★★★(4) | Unsatisfactory |
| Dairo (2016) | ★ | ★ |  | ★★ |  | ★ | ★ | ★★★★★★(6) | Satisfactory |
| Oddsson (2016) | ★ |  |  | ★★ |  | ★ |  | ★★★★(4) | Unsatisfactory |
| Voidăzan (2016) | ★ |  | ★ | ★ | ★ | ★ | ★ | ★★★★★★(6) | Satisfactory |
| Calo (2017) | ★ |  |  | ★★ | ★ | ★ | ★ | ★★★★★★(6) | Satisfactory |
| Cheruvu (2017) | ★ |  |  | ★★ | ★ | ★ | ★ | ★★★★★★★(7) | Good |
| Gilkey (2017) | ★ |  |  | ★ | ★ | ★ | ★ | ★★★★★(5) | Satisfactory |
| Ndejjo (2017) | ★ | ★ |  | ★★ | ★ | ★ | ★ | ★★★★★★★(7) | Good |
| VanWormer (2017) | ★ |  |  | ★★ | ★★ | ★★ | ★ | ★★★★★★★★(8) | Good |
|  |  |  |  |  |  |  |  |  |  |
| Degarege (2018) | ★ | ★ | ★ | ★★ | ★★ | ★ | ★ | ★★★★★★★★★(9) | Very Good |
| Ganczak (2018) | ★ |  | ★ | ★★ |  | ★ | ★ | ★★★★★★(6) | Satisfactory |
| Mansfield (2018) | ★ |  |  | ★★ |  | ★ | ★ | ★★★★★(5) | Satisfactory |
| Mendes Lobão (2018) | ★ | ★ |  | ★★ |  | ★ | ★ | ★★★★★★(6) | Satisfactory |
| Mohd Sopian (2018) | ★ | ★ | ★ | ★★ | ★★ | ★ | ★ | ★★★★★★★★★(9) | Very Good |
| O'Leary (2018) | ★ |  |  | ★★ |  | ★★ | ★ | ★★★★★★(6) | Satisfactory |
| Shapiro (2018) | ★ |  |  | ★★ |  | ★ | ★ | ★★★★★(5) | Satisfactory |
| Wang Z. (2018) | ★ |  |  | ★ | ★ | ★ | ★ | ★★★★★(5) | Satisfactory |
| Azuogu (2019) | ★ | ★ |  | ★★ |  | ★ | ★ | ★★★★★★(6) | Satisfactory |
| Lin (2019) | ★ |  | ★ | ★ | ★★ | ★ | ★ | ★★★★★★★(7) | Good |
| Shibli (2019) | ★ |  | ★ | ★★ |  | ★ | ★ | ★★★★★★(6) | Satisfactory |
| Alene (2020) | ★ | ★ | ★ | ★★ | ★ | ★★ | ★ | ★★★★★★★★★(9) | Very Good |
| Della Polla (2020) | ★ | ★ | ★ | ★★ | ★ | ★ | ★ | ★★★★★★★★(8) | Good |
| Huon (2020) | ★ |  |  | ★ |  | ★ | ★ | ★★★★(4) | Unsatisfactory |
| Lin (2020) | ★ |  | ★ | ★★ | ★★ | ★ | ★ | ★★★★★★★★(8) | Good |
| Rabiu (2020) | ★ |  |  | ★★ |  | ★ | ★ | ★★★★★(5) | Satisfactory |
| Dereje (2021) | ★ | ★ | ★ | ★ | ★ | ★ | ★ | ★★★★★★★(7) | Good |
| Destaw (2021) | ★ | ★ | ★ | ★ | ★ | ★★ | ★ | ★★★★★★★★(8) | Good |
| Humnesa (2022) | ★ | ★ | ★ | ★★ | ★ | ★ | ★ | ★★★★★★★★ (8) | Good |
| Larebo (2022) | ★ | ★ | ★ | ★★ | ★ | ★ | ★ | ★★★★★★★★(8) | Good |
| López (2022) | ★ | ★ |  | ★★ | ★★ | ★★ | ★ | ★★★★★★★★★(9) | Very Good |
| Mihretie (2022) | ★ | ★ | ★ | ★ | ★ | ★★ | ★ | ★★★★★★★★(8) | Good |
| Nguyen (2022) | ★ |  | ★ | ★★ | ★ | ★ | ★ | ★★★★★★★(7) | Good |
| Sinshaw (2022) | ★ | ★ | ★ | ★★ | ★ | ★★ | ★ | ★★★★★★★★★(9) | Very Good |
| Alaamri (2023) | ★ | ★ |  | ★ | ★★ | ★ | ★ | ★★★★★★★(7) | Good |
| Aragaw (2023) | ★ | ★ | ★ | ★ | ★★ | ★ | ★ | ★★★★★★★★(8) | Good |
| Lubeya (2023) | ★ | ★ |  | ★★ | ★★ | ★ | ★ | ★★★★★★★★(8) | Good |
| Sobierajski (2023) | ★ | ★ |  | ★★ | ★ | ★ | ★ | ★★★★★★★(7) | Good |

| **Modified Newcastle-Ottawa Scale for cohort studies** | | | | | | | | | |
| --- | --- | --- | --- | --- | --- | --- | --- | --- | --- |
|  | **Selection** | | | **Comparability** | | **Outcome** | |  |  |
| **Reference** | **Representativeness of the cohort (Maximum:★)** | **Ascertainment of exposure  (Maximum:★★)** | **Demonstration that outcome of interest was not present at start of study (Maximum:★)** | **Comparability of subjects on the basis of design or analysis (Maximum:★★)** | **Assessment of outcome (Maximum:★)** | **Follow-up long enough to occur the outcomes**  **(Maximum:★)** | **Adequacy of follow up of cohorts  (Maximum:★)** | **Total score**  **(out of 9)** | **Power** |
| Perez (2017) | ★ | ★ | ★ | ★★ |  | ★ | ★ | ★★★★★★★★★(7) | Good |
| Hirth (2019) | ★ | ★★ | ★ | ★★ | ★ | ★ | ★ | ★★★★★★★★★(9) | Very Good |
| Tatar (2019) | ★ | ★ | ★ | ★★ |  | ★ | ★ | ★★★★★★★★★(7) | Good |

**Additional file 8:** Forest plot of HPV vaccine acceptance rates by publication year


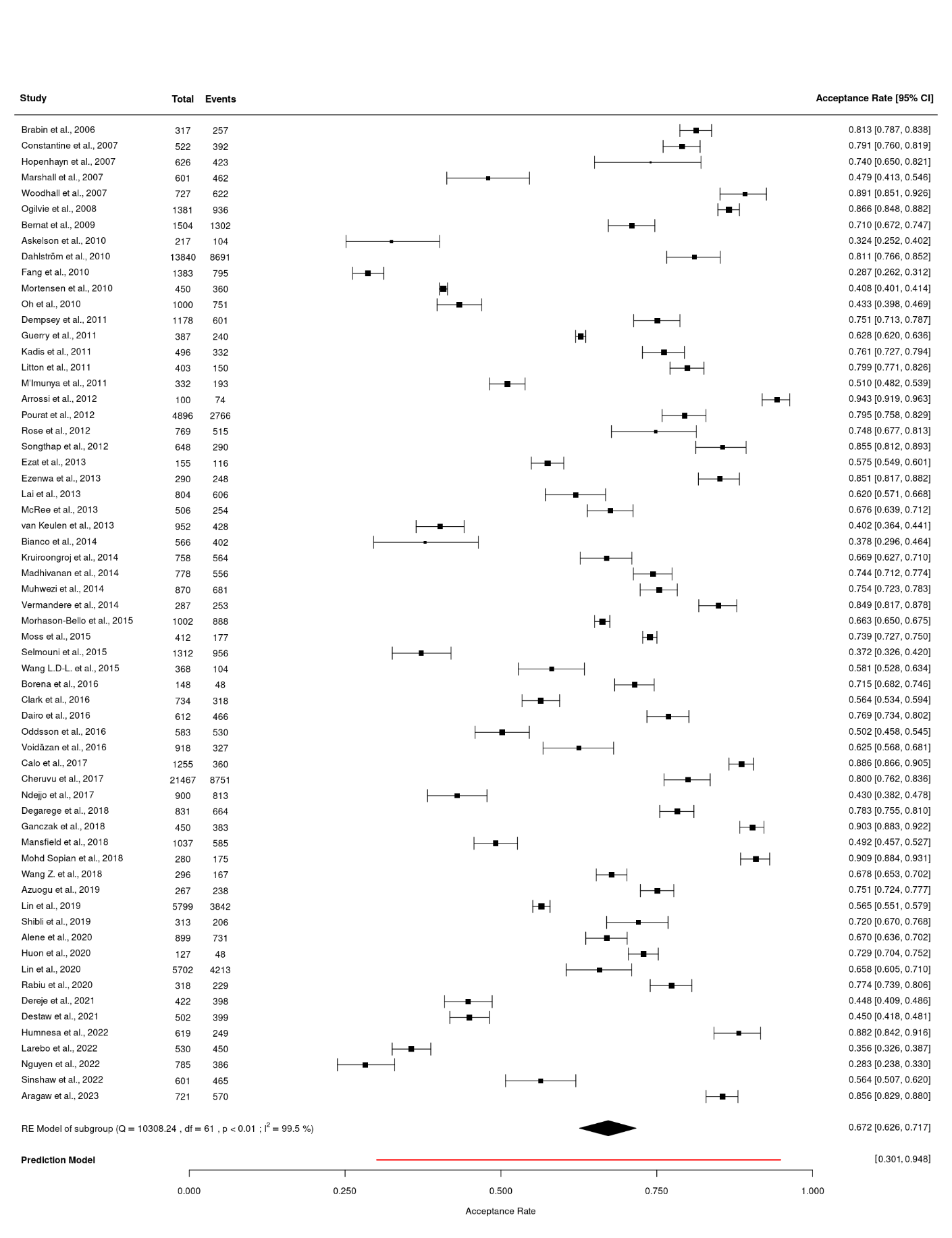
(random-effects model) (n = 62)

CI = Confidence interval

df = Degrees of freedom

Events = The number of parents/guardians in each study who reported acceptance of the HPV vaccine for their children

I² = I-squared statistic, indicating the percentage of variation due to heterogeneity

p < 0.01 = p-value, indicating statistical significance

Q = Cochran's Q statistic for heterogeneity

RE Model = Random-effects model

Total = The total number of parents/guardians in each study included in the meta-analysis

**Additional file 9**: Forest plot of HPV vaccine acceptance rates by sex of participants and world region

(random-effects model) (n = 62)

**
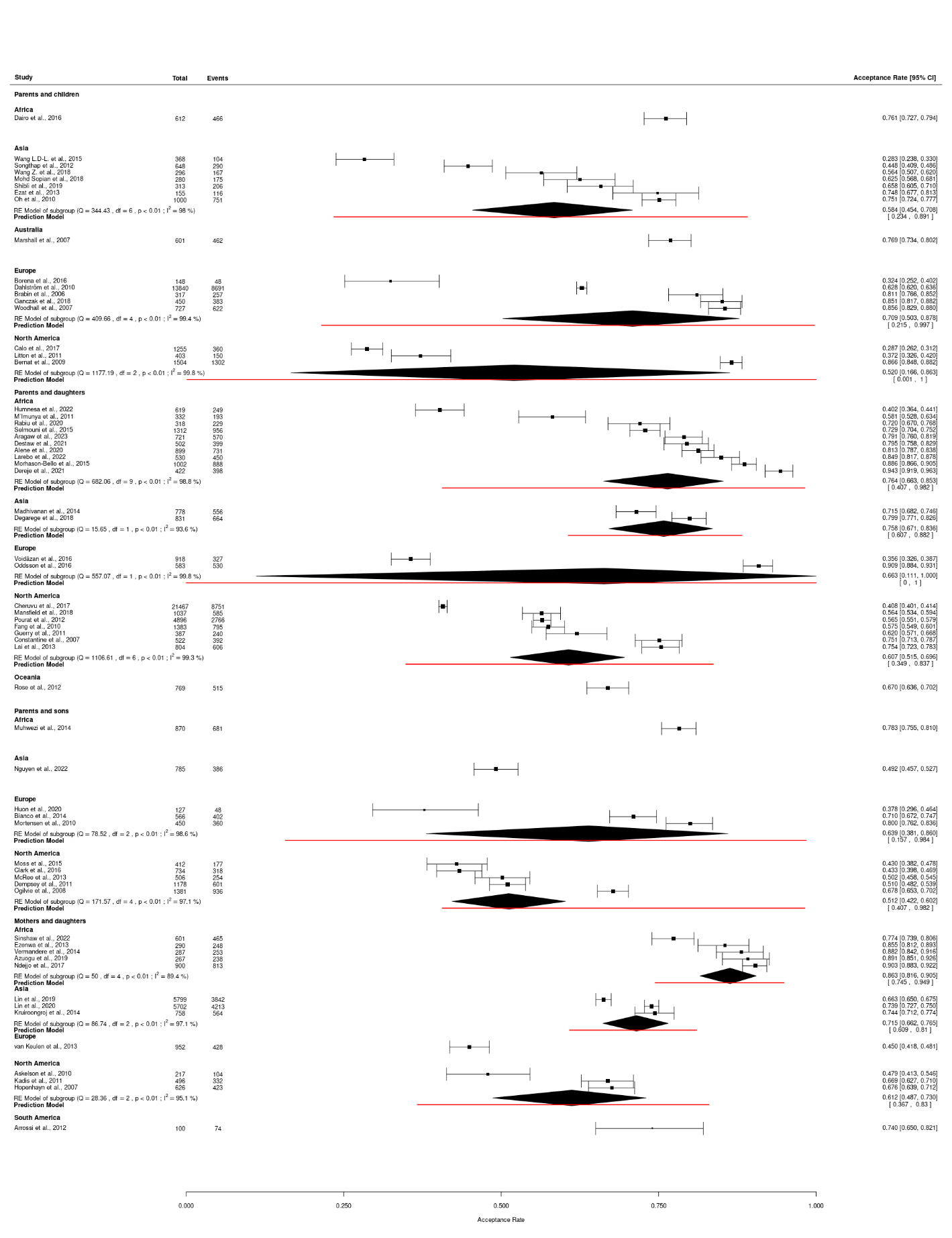
**

CI = Confidence interval

df = Degrees of freedom

Events = The number of parents/guardians in each study who reported acceptance of the HPV vaccine for their children

I² = I-squared statistic, indicating the percentage of variation due to heterogeneity

p < 0.01 = p-value, indicating statistical significance

Q = Cochran's Q statistic for heterogeneity

RE Model = Random-effects model

Total = The total number of parents/guardians in each study included in the meta-analysis
