# Supplementary figures and images for: Global parental acceptance, attitudes, and knowledge regarding human papillomavirus vaccinations for their children: a systematic literature review and meta-analysis
Source: BMC Womens Health. 2024 Sep 27;24:537. doi: 10.1186/s12905-024-03377-5 (PMC11428909; doi:10.1186/s12905-024-03377-5)

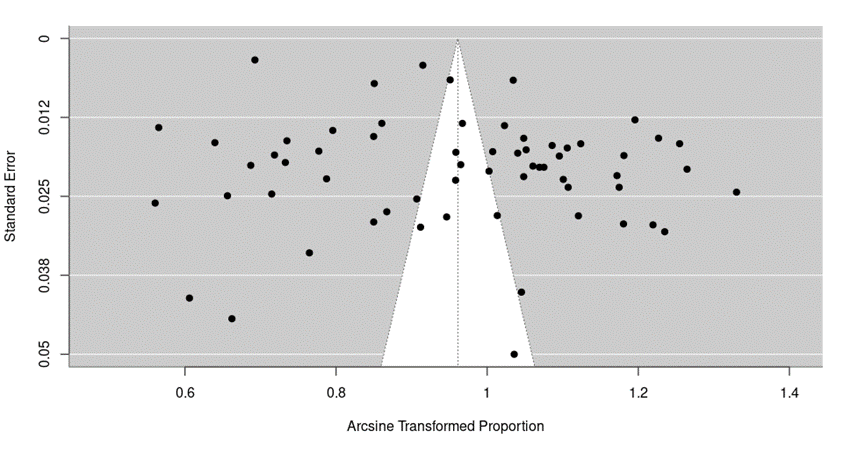

Supplement: Supplementary file 2 — Supplementary Material 2 [file 12905_2024_3377_MOESM2_ESM.jpeg]

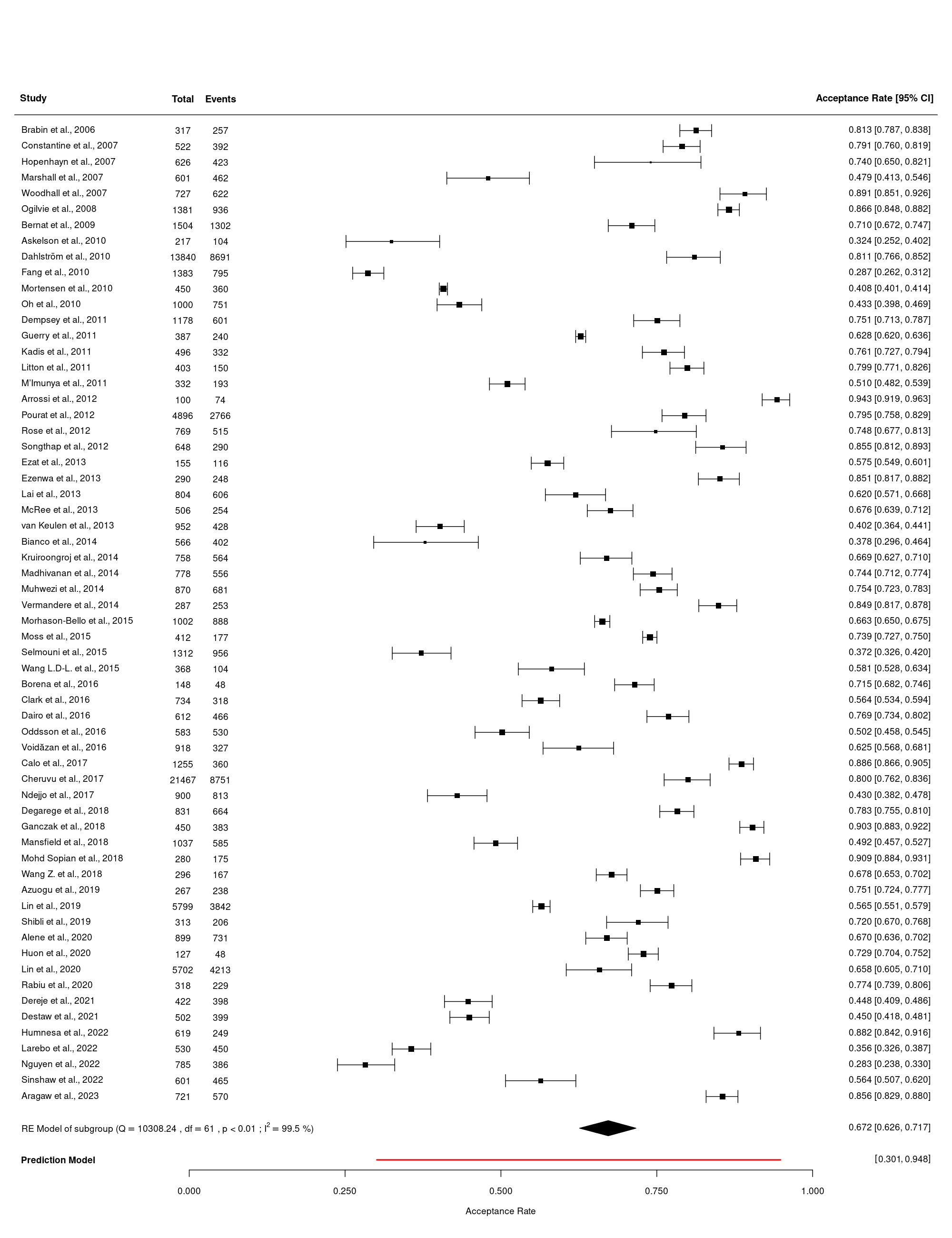

Supplement: Supplementary file 3 — Supplementary Material 3 [file 12905_2024_3377_MOESM3_ESM.jpeg]

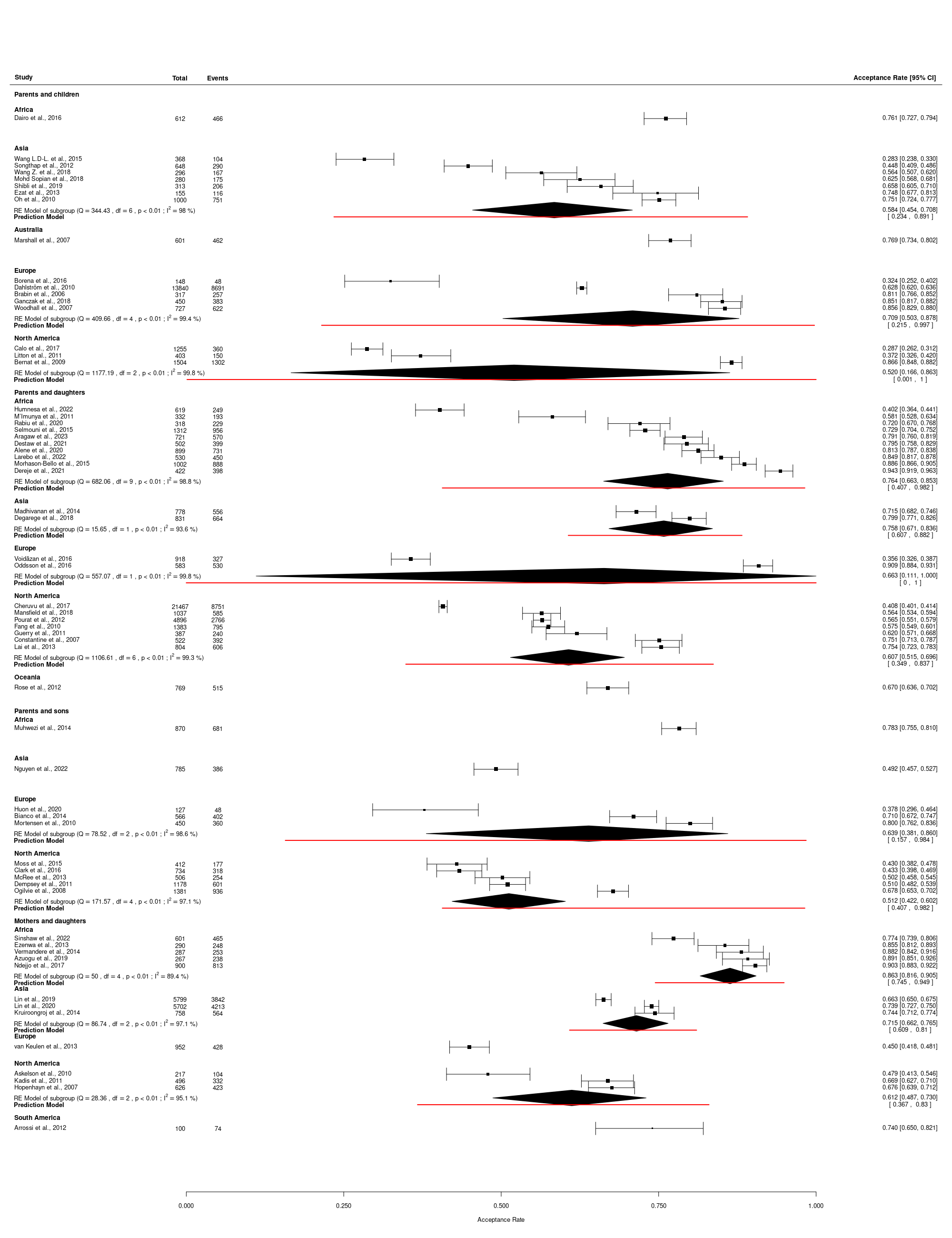

Supplement: Supplementary file 4 — Supplementary Material 4 [file 12905_2024_3377_MOESM4_ESM.jpeg]
